# Protected area staff and local community viewpoints: A qualitative assessment of conservation relationships in Zimbabwe

**DOI:** 10.1371/journal.pone.0177153

**Published:** 2017-05-19

**Authors:** Chiedza Ngonidzashe Mutanga, Never Muboko, Edson Gandiwa

**Affiliations:** 1 School of Hospitality and Tourism, Chinhoyi University of Technology, Private Bag 7724, Chinhoyi, Zimbabwe; 2 School of Wildlife, Ecology and Conservation, Chinhoyi University of Technology, Private Bag 7724, Chinhoyi, Zimbabwe; Auburn University, UNITED STATES

## Abstract

With the increase in illegal resource harvesting in most protected areas (PAs), the need to understand the determinants and relationships between PAs and local communities to enhance wildlife conservation is increasingly becoming important. Using focus group discussions and interviews, we established the determinants of PA staff-community relationship from both PA staff and local communities’ viewpoints, and assessedperceptions of their relationship with each other. The study was guided by the following main research question, ‘What is the nature of the relationship between PA staff and local communities and what are the main factors influencing the relationship?’ Data were collected through focus group discussions and interviews from four PAs and their adjacent communities in Zimbabwe between July 2013 and February 2014. Our results showed that a total of seven determinants were identified as influencing PA staff-community relationship, i.e., benefit-sharing, human-wildlife conflict, compensation for losses from wildlife attacks, communication between PA staff and local communities, community participation in the management of CAMPFIRE projects, lack of community participation in tourism in PAs, and community perceptions of PA staff or PA staff perceptions of the community. Of the seven, only one determinant, benefit-sharing, was recorded as the main factor that differentially influencesthe perceptions of community and PA staff on their relationship. Furthermore, both the communities and PA staff reported mixed perceptions on their relationship with each other. We conclude that both communities’ and PA staff’s views on determinants are largely similar in all studied PAs irrespective of PA ownership, management and/or land use. Our findings could be relevant in policy making especially in developing countries in developing PA-community relationship framework in natural resource conservation.

## Introduction

Most protected areas (PAs) have a history of human habitation before their establishment [1, 2]. For instance, many local people were evicted from their former areas of habitation when most PAs were created[3, 4] and were further prohibited from accessing natural resources that were fenced inside the established PAs [5, 6]. However, wild animals within PAs often roamed outside park boundaries, destroying crops and killing livestock and sometimes people [7–10]. The establishment of PAs was reinforced through protectionist conservation policies, later known as the ‘fences and fines’ approach or ‘fortress conservation’ [11, 12]. These policies created conflict between local people and PA staff[13, 14]. The increase in illegal resource harvestingled to a realisation that the fences and fines approach was failing as a wildlife preservation method [15, 16] and this led to the introduction of integrated conservation and development projects (ICDPs)[17, 18]. ICDPs which were reported to have gained local people support, became a popular approach for working with communities in and around PAs [19].

Some of the ICDPs which became popular through local community support in southern Africa include the Living in a Finite Environment (LIFE) in Namibia, the Administrative Management Design (ADMADE) in Zambia and the Communal Areas Management Programme for Indigenous Resources (CAMPFIRE) in Zimbabwe. In the Zimbabwean context, CAMPFIRE uses wildlife and other natural resources to promote devolution of rights to manage, use, dispose of, and benefit from natural resources to rural institutions[20, 21]. CAMPFIRE is based on the principle that, if communities receive economic benefits from wildlife, they will appreciate and contribute to its conservation [22]. Accordingly, more economic benefits are expected to accrue to communities when they have higher conservation ethics, for example, if communities desist from poaching, more animals will be available for hunting which will eventually mean more revenue for the communities. Evidence from some areas in Zimbabwe shows that poaching was rampant prior to CAMPFIRE [23], but its introduction in the late 1980s resulted in the decline of poaching in some areas [24]. Benefits from CAMPFIRE helped to promote positive relationships between PA staff and local communities [25]. In this study, positive PA-community relationship means PA staff and the local community interact well and tolerate each other. However, CAMPFIRE still remains with a number of challenges including the bouncing back of poaching in some areas just after a few years of its introduction [20, 23].

Earlier studies have looked different aspects of PA staff-community relationships, e.g., human-wildlife conflicts (HWC) and benefit-sharing [26–29], communication between PA staff and communities [30, 31], collaborative management [32, 33], communities attitudes [26], and PA staff attitudes towards communities [25]. However, few studies evaluate PA-community relationships between different conservation areas and tenure regimes. For example, Simelane et al. [34] investigated PA-community relationships using five national parks in South Africa and Tessema et al. [26] used four PAs in Ethiopia. Moreover, there is an observed tendency in the literature to study PA-community relationships usingonlythe community’s viewpoint[35, 36, 37], with very few studies analysing both PA staff and community perceptions [38]. These studies have highlighted significant differencesin the perceptions of PA staff and communities. For example, while PA staff in Samburu and Buffalo Springs National Reserves in Kenya reported that they sufficiently initiated and maintained dialogue with their adjacent communities, the communities reported that communication with PA staff was limited and irregular [38]. Furthermore, in the same study, while PA staff perceived the benefits the communities got from PAs as satisfactory and sufficient, the communities were unsatisfied with the small percentage of community members employed by the park, and the amount of revenue-distribution between the parks and the communities where communities only got a very small percentage [38]. These differences in PA staff and community perceptions indicate the need for region or country specific studies to assess PA-community relationships if stakeholder concerns are to be addressed in order to identify potential problem areas regarding PA management and wildlife conservation[39].

A knowledge gap exists in Zimbabwe considering that studies on PA-community relationships in the country have been done on single PAs and using only community’s viewpoints, e.g., Mhlanga [40] looked at conflict between wildlife and people in Kariba, and Mombeshora and Le Bel [41] assessed parks-people conflicts in Gonarezhou National Park. A recent attempt to comprehensively study conservation relationships from both PA staff’s and local communities’ perspectives is that of Mutanga et al. [42] who quantitatively assessed conservation relationships from 1,071 people from four PAs and adjacent communities. However, that study did not consider the heterogeneity that exist among community members and PA staff in different PAs hence it groups together all communities and all PA staff. This present study contributes to the PA-local community relationship literature through examining the determinants of conservation relationships and PA-community relationships from both the PA staff and local communities’ viewpoints while taking into consideration the different communities and PAs, as well as sub-groups within communities to allow for an exploration of different experiences among community members. By understanding how PA staff and communities perceive the magnitude and value of each determinant in influencing PA-community relationships, PA management can effectively address relevant stakeholder needs and minimise conflicts between PA staff and adjacent communities. Moreover, the study compares these relationships under different management regimes in Zimbabwe. The study was guided by the following main research question, ‘What is the nature of the relationship between PA staff and local communities and what are the main factors influencing the relationship?’ The specific objectives of the study were: (1) to establish and compare the determinants of PA staff-community relationship across different ownership and management regimes, (2) to assess the kind of influence each determinant has on PA-community relationships, and (3) to compare PA staff-community perceptions of their relationship.

## Materials and methods

### Study sites

**Fig 1:**
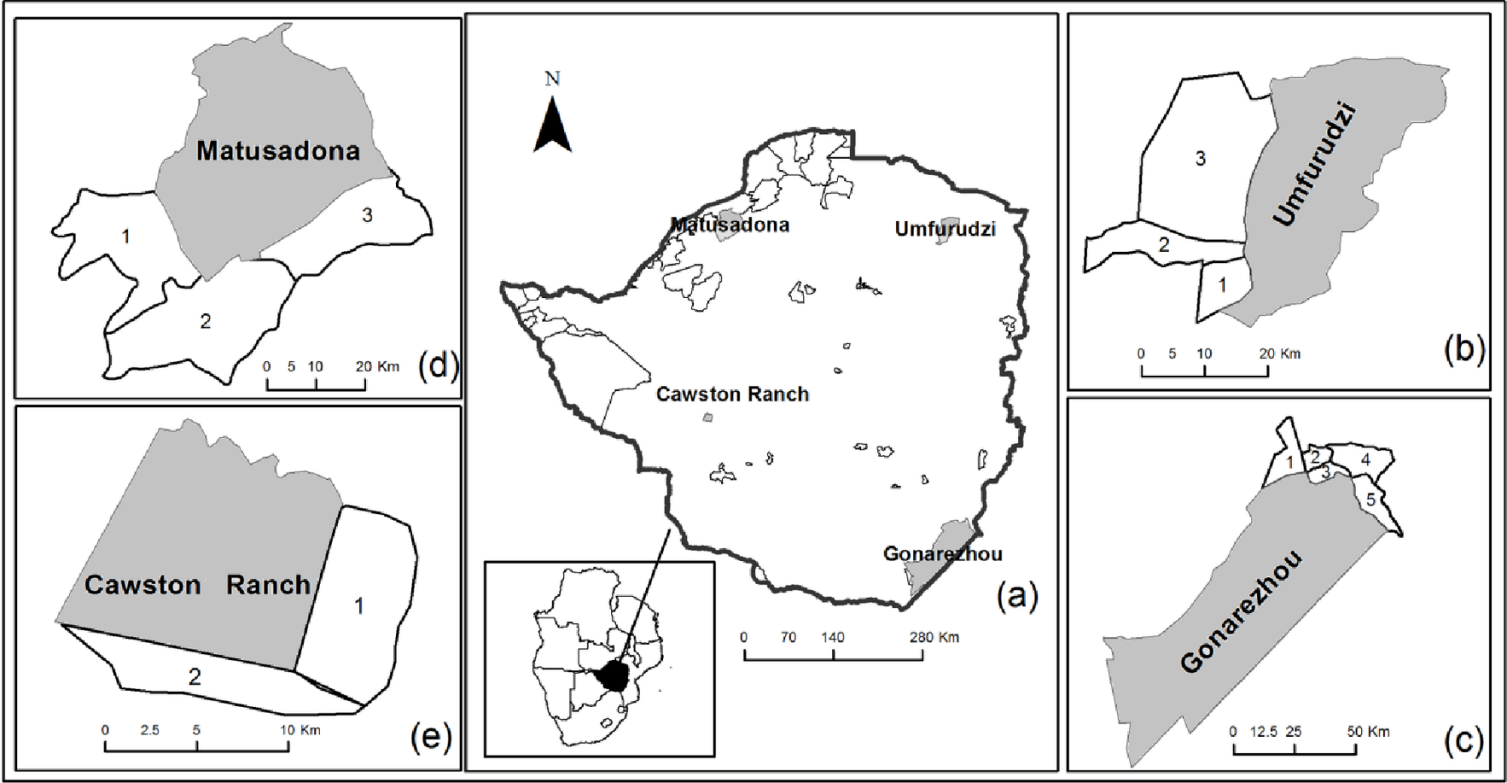
Location of the four study sites in Zimbabwe(See also Table 1 for details).

Four study sites were selected purposively to give a broad understanding of PA-community interactions in Zimbabwe. To select the PAs, we considered ownership (both state ownership and private ownership) and type of management (i.e., publicly managed, privately managed or managed by a public-private partnership), land usepatternsof the PA (national park or safari area), as well as whether the adjacent communities did or did not have CAMPFIRE. A national park is mandated for conservation through non-consumptive utilisation and therefore trophy hunting is not allowed. In a safari area controlled trophy hunting is permitted within the park and such trophy hunting is controlled through a quota system that aims to promote sustainable hunting. The four selected study sites were: Umfurudzi Park, Gonarezhou National Park, Matusadona National Park and Cawston Ranch, and their surrounding communities (Fig 1; Table 1). Although Umfurudzi Park is gazetted as a safari area, trophy hunting was temporarily suspended due to the population decline and local extinction of some species. All the sampled villages surrounding a PA are referred to as a community in this study, hence we have four communities: Umfurudzi, Gonarezhou, Matusadona and Cawston Ranch. While we acknowledge that there may be spatial and socio-economic differences between these villages, we grouped together all villages adjacent to a PA into one community because we wanted a more general outlook of PA staff-community relationships.

**Table 1:** General characteristics and organisation of the four PAs and their surrounding communities.

Attributes	Study site			
	Umfurudzi	Gonarezhou	Matusadona	Cawston Ranch
Status	Safari Area	National Park	National Park	Safari Area
Ownership	Government	Government	Government	Private
Management	Public-private partnership	Public-private partnership	Public	Private
Year established	1981	1930 as a Game reserve, upgraded to a National Park in 1975	1963 as a Game reserve, upgraded to a National Park in 1975	1988
Size (km^2^)	760	5,053	1,400	128
Forms of tourism	Photographic, sport fishing	Photographic, sport fishing	Photographic, sport fishing	Trophy hunting
Study areas (as depicted in Fig 1)	1- Sanye, 2-Mufurudzi 1, and 3-Mufurudzi 2	1-Chizvirizvi, 2-Mupinga, 3-Chitsa, 4-Mutandahwe, and 5-Mahenye	1-Nebiri, 2-Musambakaruma 2, and 3-Musambakaruma 1	1-Ward 10 and 2-Ward 9.
Sources of community livelihoods	-Small-scale subsistence and cash crop farming -Small scale livestock production -Gold panning	-Small-scale substance and cash crop farming -Small scale livestock production	-Small scale subsistence and cash crop farming -Very little livestock production due to tsetse fly prevalence	-Small-scale subsistence and cash crop farming -Small scale livestock production
CBNRM projects	None	CAMPFIRE	CAMPFIRE	None
*Estimation of PA benefits to communities*				
Monetary benefits from PAs*	None	None	None	None
Non-monetary benefits from PAs	-Ecosystem services, e.g., flood control, fruits and clean air, casual workers are sourced from the local communities, few permanent employees are sourced from the communities	-Ecosystem services, controlled harvesting of thatching grass and firewood, controlled livestock grazing especially during drought, casual workers are all sourced from the local communities, few permanent employees are sourced from the communities, access to cultural and traditional sites	-Ecosystem services, casual workers are all sourced from the local communities, few permanent employees are sourced from the communities, access to cultural and traditional sites	-Ecosystem services, controlled harvesting of thatching grass, casual workers are sourced from the local communities, few permanent employees are sourced from the communities
Monetary benefits from CAMPFIRE per household	na	Head tax (usually about US$1 per year)**	Head tax (usually about US$1 per year)**	na
Collective benefits from CAMPFIRE	na	Include: schools, grinding mills, boreholes***, hardware store, trucks	Include: schools, grinding mills, boreholes, clinics	na

Notes: CBNRM = Community-Based Natural Resource Management; CAMPFIRE = Communal Areas Management Programme for Indigenous Resources

*Information on income generated by each PA was difficult to access during the study

**In the past there were more monetary benefits per household but now people only benefit from the head tax

***a borehole is a hole drilled in the ground to extract water

na means not applicable.

### Data collection

#### Community perceptions of determinants and PA staff-community relationship

Data were collected using focus group discussions (FGDs) and in-depth interviews (S1 Table and S2 Table) between July 2013 and February 2014 as explained below. FGDs were used to establish communities’ perceptions of the determinants of PA staff-community relationship and of their relationship with PA staff. To establish the determinant factors of their relationship, respondents were asked to detail their expectations and explain whether the expectations were met or not. FGDs were guided by three main open ended questions meant to solicit more responses from the people:(1) what are your expectations from PAs and PA staff? (2) Explain whether your expectations are being met or not, and (3) how do you describe your relationship with the PA and why?Due to the exploratory nature of the study, we had no pre-determined list of answers and as such, all the determinants discussed in this paper were instigated from these FGDs. However, where a focus group did not mention certain issues raised by former group(s), a follow-up question(s) was raised in that regard. The instrument was piloted among local people in Umfurudzi, through two FGDs, one with ten males and the other with ten females, all from Magazi village adjacent Umfurudzi Park. This village (Magazi), although it was part of the relevant population, was excluded from the final sampleto exclude any chances of peer influence of other participants [43].

Ten discussants were targeted for each FGD (Table 2) as recommended by. Purposive sampling guided the initial selection of focus group discussants. Purposive sampling groups participants according to preselected criteria relevant to a particular research question [44]. Prior to purposive selection of FGDs participants, a formal request was made through community traditional leaders where ten participants were selected per FGD. FGDs participants were clustered according to their roles in the community. Four FGDs were held in each community, the first FGDs comprised community leaders, i.e., Chiefs, Village Heads and/or Counsellors, the second FGDs comprised male headsof the families, the third FGDs comprised females with families while the final FGDs comprised unmarried young people (18–35 years). A total of 16 FGDs were held with 160 people (40 from each community) comprising 104 (65%) males and 56 females (35%) participating. Overall, 27 (17%) respondents had no formal education, 81 (51%) respondents had primary level education (Grade 0–7), 39 (24%) respondents had secondary education (Form 1 –Form 4 or 6)and 13 (8%) respondents had tertiary education (college diploma, undergraduate degree or above).

**Table 2:** Distribution of FG discussants among community members.

Area	District	Ward	Population	Estimated number of households	Average household size	Distribution of FG discussants			
						Community leaders	Male heads	Females with families	Youths
*Umfurudzi*									
Sanye	Shamva	27	3 640	731	5.0	3	3	4	3
Mufurudzi 1	Shamva	16	7 380	1 614	4.6	4	3	3	3
Mufurudzi 2	Shamva	14	3 853	800	4.8	3	4	3	4
*Total*				3 270		10	10	10	10
*Gonarezhou*									
Chizvirizvi	Chiredzi Rural	22	6 331	1 378	4.6	-	4	-	3
Mupinga	Chiredzi Rural	4	5 651	1 305	4.3	-	3	-	4
Chitsa	Chiredzi Rural	5	4 366	986	4.4	-	3	-	3
Mutandahwe	Chipinge Rural	29	12 949	2 450	5.3	5	-	5	-
Mahenye	Chipinge Rural	30	3 671	707	5.2	5	-	5	-
*Total*				6 749		10	10	10	10
*Matusadona*									
Nebiri	Kariba Rural	7	1 633	385	4.2	2	2	2	2
Nebiri	Kariba Rural	8	5 768	1 165	5.0	3	3	3	3
Musambakaruma 1	Kariba Rural	9	2 999	640	4.7	2	2	2	2
Musambakaruma 2	Kariba Rural	10	1 564	349	4.5	3	3	3	3
*Total*				2 395		10	10	10	10
*Cawston Ranch*									
Ward 9	Umguza	9	5 626	1 411	4.0	5	5	5	5
Ward 10	Umguza	10	2 887	607	4.8	5	5	5	5
*Total*				1 950		10	10	10	10

Divisions into sub-groups for FGDs was done to ensure homogeneity among discussants so as to maximise disclosure and to allow for an exploration of different experiences considering that different groups of people have different roles in the community. Community leaders usually make overall decisions for their communities. They therefore have knowledge of what is generally happening in the communities, whether people are happy or not and what makes them happy or not happy. In the communities studied, the male heads usually have decision-making powers for their households and are expected to carry the social and economic responsibility for the well-being of household members. They usually work in agricultural wage labour and cash crop production [45]. Women are usually responsible for caring and feeding the children, and engaging in jobs such as working on farms, gardening, and doing household chores, including domestic water and firewood collection [45]. Finally, the youth are involved in projects like handcrafts, vegetable growing and home improvement. However, young people are often isolated and unable to get involved in many community development activities [46]. The male heads and youths (males) are often involved in bush meat hunting for subsistence and for sale. All FGDs were held at convenient places within the study communities. FGDs were facilitated by the moderator, i.e., the first author, with the help of a trained observer carefully chosen from the communities. All conversations (FGDs and interviews) were electronically recorded and we also took down notes for back up. Discussions lasted between 60 and 90 minutes.

Table 2 shows the distribution of community members who participated in FGDs and the estimated population of community members in a district(population statistics were derived from. The districts are comprised of wards divided into villages and then households within each village. A ward is made up of six or seven villages [47].

#### PA staff perceptions of determinants and community-PA staff relationship

Purposive sampling was used to select interviewees from PAs. The managers (or supervisors in the absence of a manager) on duty in the PAs at the time of data collection were purposively selected. The main goal of purposive sampling was to glean knowledge from individuals that were more knowledgeable about PA mandates versus reality with regards to PAs-community interactions and relationships were managed [48]. These would best enable us to answer our research questions. Because not more than one manager was available in each of the four PAs during the time of data collection, we ended up interviewing rangers also, and we grouped them as PA staff. Thus, using the snowballing method, the purposively selected interviewees were asked to identify additional potential interviewees [49], who were assumed to have extensive knowledge on the PAs’ relationships with neighbouring communities especially those who had worked in the PA for a long time. With the exception of Matusadona National Park where three interviews were carried out with the PA staff, four interviews were carried out with PA staff in the other three PAs, giving a total of 15 interviews. The sample comprised of 86.7% (n = 13) males and 3.3% (n = 2), i.e., Umfurudzi (3 males and 1 female); Gonarezhou (4 males, 0 female); Matusadona (3 males and 0 female), and Cawston Ranch (3 males and 1 female). Overall, 40% (n = 6) intervieweeshad primary level education (Grade 0–7), whereas 60% (n = 9) had tertiary education (college diploma, undergraduate degree or above). Four interviewees had been working in the PAs for less than five years, one had between six and 10 years of working in the PA, whereas 10 had more than 10 years experience of working in the PAs. Interviews took place at the respective PAs and interviews lasted between 30 and 45minutes. The interviews were guided by three questions:(1) what are your expectations from communities? (2) Explain whether your expectations are being met or not, and (3) How do you describe your relationship with the community and why?

#### Ethics statement

All participants gave their verbal and informed consent to participate in the study after they were verbally read all the elements of written consent. Verbal consent was considered appropriate over written consent considering that the procedures involved no risk and because information such as names or other identifiers was not recorded. The research procedures which includes ethics issues were approved by Chinhoyi University of Technology Research Committee.

### Data analysis

Following, qualitative data from both FGDs and interviews were analysed using content analysis where the key issues were grouped into themes. A thematic coding framework was designed based on the emerging themesand themes were colour coded using Microsoft Word. The internal coherence of the defined set of codes was checked by asking two other researchers (colleagues) to use them to code the same focus group discussions and interviews. The discrepancies were very minor which indicated that the codes were coherent and unambiguous, and were defined precisely enough [50]. After coding, a text file was generated for each code that listed the relevant data. Careful, systematic analysis of these text files generated a rich description of the PA staff-community relationship for each study site[37]. A comparative approachwas used where PA staff and local community views of their relationship and the determinant factors were compared.

Determinants of PA staff-community relationships were established based on expectations on different issues mostly mentioned by focus group discussants and interviewees. A qualitative analysis approach was used to group the main expectations into themes where each theme represented a determinant. Focus group discussants and interviewees’ responses were sorted into different influencing determinants. Determinants were created inductively for each group within each community and for each PA after consideration of the responses gathered from the FGDs and interviews [36]. This approach allowed us to capture qualitative explanations of specific determinants thus classifying them into categories which enabled easy comparisons among the: (i) different groups within the same community, (ii) different communities, and (iii) PAs and communities. The categories were assigned based on community benefits received, human-wildlife conflict, compensation for losses from wildlife, communication between PA staff and local communities, community participation in the management of CAMPFIRE projects, community participation in tourismin PAs, wildlife conservation problems caused by community members, community perceptions of PA staff, and community perceptions of the management of PAs.

PA staff and local community perceptions of their relationship were classified into negative or positive based on level of interaction between the two parties. Negative relationship mean PA staff and community members have undesirable (bad) interaction and positive relationship mean PA staff and community members have derisible (good) interaction. Bad interaction mean there is no or there is low interface between PA staff and adjacent community, and good interaction mean there is high interface.

In addition, to content analysis we conducted an objective analysis where we analysed people’s expectations versus reality, i.e., whether their expectations have a base or whether they are in sync with the objectives of both PAs and CAMPFIRE.

## Results

### Community perceptions of determinants and PA staff-community relationship

Based on FGDs on community expectations from PAs, seven determinants of communities’ relationships with PA staff emerged, i.e., benefit-sharing, human-wildlife conflict, compensation for losses from wildlife attacks, communication between PA staff and local communities, community participation in the management of CAMPFIRE projects, lack of community participation in tourism in PAs, and community perceptions of PA staff (Table 3). The community had many expectations from PAs such as grazing land and compensation for losses from wildlife depredation in Umfurudzi and Gonarezhou, employment (where a greater percentage of PA staff would come from the communities) in all communities, openand sufficient communication between the PAs and communities in all communities, and being consulted on decisions that impacted them. Many of these expectations were not being met and contributed to the reasons forthe negative PA-community relationships in all the four communities.

**Table 3:** Determinants of PA-community relationships based on communities’ expectations.

Determinant	Expectations	Current status			
		Community leaders	Male heads	Females with families	Youths
Benefit-sharing	Employment; construction of dams, boreholes, schools, roads, electricity, and hospitals; game meat; thatching grass; grazing land ^(U, G, M & C)^	Majority views:Very few benefits ^(U)^; limited use of natural resources mainly thatching grass ^(G)^, limited benefits from CAMPFIRE e.g., boreholes ^(G & M)^; low level of employment^(G, M & C)^;limited other benefits e.g., workshop services and transport^(C)^ Minority views: Use of natural resources permitted especially for leaders like chiefs ^(G & C)^, considerable benefits from CAMPFIRE ^(G & M)^, other benefits like workshop services available to many people ^(C)^	Unanimous views: No benefits ^(U)^; Limited use of natural resources mainly thatching grass ^(G)^, limited benefits from CAMPFIRE e.g., boreholes ^(G & M)^; low level of employment^(G, M & C)^; a number of other benefits e.g., workshop services and transport but leaders got preferential treatment ^(C)^	Unanimous views:Same responses as from male heads for all communities	Unanimous views:Same responses as from male heads for all communities
Human-wildlife conflict	Effective problem animal control measures ^(U, G, M & C)^	Unanimous views: High HWC, park officials take long to respond to complaints ^(U, G, M & C)^	Unanimous views: High HWC^(U, G, M & C)^	Unanimous views: High HWC^(U, G, M & C)^	Unanimous views: High HWC^(U, G, M & C)^
Compensation for losses from wildlife	Monetary compensation for crop damage, or livestock depredation by wildlife^(U, G, M & C)^	Unanimous views: None^(U, G, M & C)^	Unanimous views: None^(U, G, M & C)^	Unanimous views: None^(U, G, M & C)^	Unanimous views: None^(U, G, M & C)^
Communication	Open and efficient communication^(U, G, M & C)^	Unanimous views: Bad^(U)^, Limited to community leaders ^(G, M & C)^	Unanimous views:Nonexistent^(U)^, Limited to community leaders ^(G, M & C)^ Majority views: informal and irregular ^(G, M & C)^ Minority views: Not open ^(G, M & C)^	Unanimousviews:Non existent^(U)^, Majority views: Limited to community leaders ^(G, M & C)^ Minority views:informal and irregular	Unanimousviews:Non existent^(U)^ Majority views: Not open ^(G, M & C)^ Minority views: informal and irregular
Participation in PA tourism management	Recognition of traditional knowledge; participate and receive benefits from tourism^(U, G, M & C)^	Unanimous view:No involvement ^(U, G, M & C)^	Unanimous view:No involvement^(U, G, M & C)^	Unanimous view:No involvement^(U, G, M & C)^	Unanimous view:No involvement^(U, G, M & C)^
Collaborative participation in CBNRM management	To be involved in more important decisions in CAMPFIRE like revenue sharing decisions^(G & M)^	Unanimous view: Only a few are partly involved ^(G & M)^ Majority view: Although involved, the communities’ views are not taken into consideration	Unanimous view: Only a few are partly involved ^(G & M)^ Minority view: Although involved, the communities’ views are not taken into consideration	Unanimous view: Only a few are partly involved ^(G & M)^	Unanimous view:The youths are not involved ^(G & M)^
Perceptions of PA staff	PA management to be more sensitive to community needs, respond quickly to calls for problem animals and to consult and value community input ^(U, G, M & C)^	Unanimous view:Not caring, e.g., the erection of the fence boundary ^(U & G)^, Not considerate ^(C)^, take long to respond to complaints ^(U, G, M)^ Minority view: They relate well with communities^(U, G, M & C)^	Unanimous view:Not caring, e.g., the erection of the fence boundary ^(U & G)^, late to respond to complaints ^(U, G, M)^, do not teach the community to participate in tourism^(U, G, M & C)^, Not considerate ^(C)^	Unanimous view:Same responses as from male heads for all communities	Unanimous view:Same responses as from male heads for all communities

Symbols in superscript form represent names of communities, that is, Umfurudzi community (^U^), Gonarezhou community (^G^), Matusadona community (^M^) and Cawston Ranch community (^C^). Where a symbol for a particular community is present indicates the community which raised the issue(s).

The most common indicators of unmet expectations across all PAs and focus groups were unsatisfactory benefits from the PAs for example, lack of access to grazing land and water for livestock due to the boundary fence erected in Umfurudzi Park and Gonarezhou National Park and human-wildlife conflict. A villager from the male focus group in Umfurudzi community had this to say:

“They brought their cheetahs here. Now four of my cattle were killed. As if that is not enough, our crops, especially those of us who are close to the boundary, are always destroyedby the kudus and sables. The worst part is that up to today, I have not been given even a single cent for my losses”.

Concerning grazing land, a village head from Gonarezhou National Park lamented:

“They fenced the park, now we no longer have grazing land or water for our cattle. Our cattle are dying in numbers”.

In the same community, a woman from another focus group explained:

“The Park erected the fence without even consulting us, our children used to go to school because we would sell the cattle to get money for school fees, but now they no longer go to school. In times of hunger, we would sell the cattleand use the money to buy food. Now because of this fence, our cattle are dying and those that are still alive are so thin that nobody wants to buy them. How then are we supposed to live?”

From Matusadona community, dissatisfaction with benefit sharing mainly arose from decreasing benefits from CAMPFIRE:

“When CAMPFIRE started, we used to benefit a lot in form of cash, ward offices, schools and many other things, but now we are not getting anything, the council is the only one benefiting. Actually, getting money from CAMPFIRE has become a thing of the past. We are in the second year now without getting a single cent but the hunters are still coming as before”, (villager, male focus group).

While some communities around Gonarezhou and Matusadona National Parks had CAMPFIRE, those in Umfurudzi Park do not have a similar privilege:

“We hear about this thing called CAMPFIRE, but we do not have it here. As such the park does not benefit us in any way. If the park would at least, build us schools, roads, dams and help us with electricity we would be very grateful”, (villager in the male focus group).

The issue of employment was another source of dissatisfaction in all communities:

“Very few people from our community are employed in the ranch; they prefer people from far away. At the end of the day, one has to do what one has to do to survive. Those animals are our only means of survival”, (boy in the youth focus group in Cawston Ranch).

However, as communities are heterogeneous, not everyone shared the same opinions. For example, while many villagers were not happy with the benefit sharing, a few were content. For example one of the leaders in Cawston Ranch had this to say:

“They help us with a vehicle when we have important journeys, e.g., during illnesses or funerals and they also help us with a tractor for ploughing our fields.”

In the same vein, a village head from the community leaders’ focus group in Gonarezhou pointed out:

“CAMPFIRE helps us a lot. Besides communal benefits like grinding mills, hardware store, truck and tractor, people enjoy individual benefits like meat from the hunted elephants and occasional cash dividends”.

Majority of the focus group discussants in all communities reported negative perceptions on their relationship with PA staff. Explanations given mainly revolved around those aspects where communities expressed much dissatisfaction especially due to expectations not met. These include: (i) no interaction between the PAs and the adjacent communities; (ii) PAs were not concerned about the communities’ welfare, e.g., presence of boundary fences in some parts of Umfurudzi Park and Gonarezhou National Park led to restriction in livestock grazing; (iii) no/delayed response to human-wildlife conflicts, and (iv) limited benefits from PAs (Table 3). Similarly, the minority (all of whom were community leaders) who reported positive perceptions on their relationship with PA staff expressed satisfaction with some of their expectations which were being met. For example, during the community leaders’ focus group discussion in Matusadona, one counsellor had this to say:

“Although they often take long to respond to complains, PA staff are cheerful and they relate well with us. We drink beer together in beer halls and they even come to our homes for beer when they are free”.

While communities have diverse expectations, not all of their expectations are the responsibility of PAs. The CAMPFIRE, or any other CBNRM projects as well as other institutions like the Local Government and Non-Governmental organisations also have an important role to play. PAs are certainly expected to provide some of the services like employment and conservation awareness programmes. However, many of the communities’ expectations, for example, infrastructural development are beyond the mandate of PAs. More so, some of the expectations like grazing land for livestock and harvesting of thatching grass (Table 4), may, if not carefully planned or managed, go against what PAs stand for since their main objective is biodiversity conservation.

**Table 4:** Responsibilities of different institutions with regards to benefit provision to communities. Notes: ‘√’ indicates that the respective authority is responsible for providing that benefit, ‘?’ indicates that the respective authority may provide the benefit if it is possible, ‘X’ indicates that it is not the responsibility of the respective authority to provide that benefit although it may if it deems fit.

Community expectation	PA	CAMPFIRE	Other institutions like Local Government Agencies or Non-Governmental Organisations (NGOs)
Employment	✓	✓	✓
Water provision	?	✓	✓
Schools	X	?	✓
Hospitals	X	?	✓
Electricity supply	X	?	✓
Livestock grazing	?	✓	✓
Thatching grass	?	✓	✓
Roads	X	?	✓
Vehicles for Transport	X	?	✓
Tractors for ploughing in the fields	?	?	✓
Conservation awareness programmes	✓	✓	✓
Skills development workshops, e.g., in tourism	x	✓	✓

### PA staff perceptions of determinants and community-PA staff relationship

Seven determinants of PA staff relationships with the local communities emerged from PA staff expectations for and from the communities that were derived during interviews, i.e., benefit-sharing, human-wildlife conflict, compensation for losses from wildlife attacks, communication between PA staff and communities, collaborative participation in CAMPFIRE management, collaborative participation in PA tourism management, and PA staff perceptions of the community (Table 5). PA staff had expectations for the communities, for example, in all the four PAs, PA staff expected that adjacent communities had to benefit from their neibouring PAs. On top of this, PA staff in all the four communities also expected adjacent communities to attend all conservation training workshops or awareness campaigns organised for them, where they are taught on the importance of conserving nature. Communities are therefore encouraged and are expected to desist from indulging in illegal activities that have negative impacts on conservation like poaching, encroachment, illegal harvesting of thatching grass and firewood collection among others. Furthermore, when they have grievances or are unhappy about something, PA staff expected communities to communicate their grievances using the right channels, that is going through their community leaders in a peaceful way. However, although communities expected this from communities, meeting these expectations is not necessarily a pre-requisite for benefit-sharing. One interviewee from Gonarezhou National Park commented,

“The communities are totally unpredictable you know, one day you think you are together, they are all supportive, the next day they are totally against you, you organise a workshop for them, they don’t come. However, giving them controlled access to some wildlife resources like thatching grass, whenever we can is part of our social responsibility, it does’t matter whether they meet these expectations or not”.

**Table 5:** Determinants of PA-community relationship based on PA staff expectations.

Determinant	Expectations	Current status
Benefit-sharing	Holding capacity building workshops for the local community^(U, G, M & C)^; employment^(U, G, M & C)^; improve infrastructure^(U)^; allow limited access to the use of wildlife resource; and CAMPFIRE benefits ^(G & M)^; transport, subsidised game meat, tractors, water, and workshop services among other benefits ^(C)^	Unanimous views:Fewbenefits for communities^(U)^; Limited access to the use of wildlife resource like thatching grass^(G)^, CAMPFIRE benefits ^(G & M)^; employment ^(G, M & C)^, transport, subsidised game meat, tractors, water, and workshop services ^(C)^, conservation awareness campaigns^(U, G, M & C)^
Human-wildlife conflict	Reduce human-wildlife conflict ^(U, G, M & C)^	Unanimous views:Erection of the fence boundary^(U, G)^; tightening problem animal control measures ^(U, G, M & C)^
Compensation for losses from wild animals	Partly compensate the community for their losses^(U, G, M & C)^	Unanimous view:None^(U, G, M & C)^
Communication between PA staff and local communities	Open and sufficient communication^(U, G, M & C)^	Unanimous views:Not regular and limited ^(U, G & M)^; Scheduled meetings with community leaders ^(C)^
Community participation in the management of CAMPFIRE projects	Community to be involved in decision making for CAMPFIRE ^(G & M)^	Unanimous views:Limited involvement in CAMPFIRE management ^(G & M)^
Community participation in tourism in PAs	Enhance community participation and benefits from tourism^(U, G, M & C)^	Unanimous views:Community not involved ^(U, G, M & C)^
Problems caused by the community	Communities to stop poaching and encroachment^(U, G, M & C)^	Unanimous views:Communities involved in illegal hunting^(U, G, M & C)^; human encroachment ^(U, G)^; mining ^(U, G)^

Symbols in superscript form represent names of PAs, that is, Umfurudzi (^U^), Gonarezhou (^G^), Matusadona (^M^) and Cawston Ranch (^C^). Where a symbol for a particular community is present indicates the community which raised the issue(s).

According to PA staff, some of these expectations, for example, community benefits from PAs (in Gonarezhou, Matusadona and Cawston Ranch) were met but to a very less extent. According to one interviewee from Gonarezhou National Park, harvesting of thatching grass is only done during the rainy season and is strictly controlled and monitored. Because there are many families who are in need of thatching grass (see Table 2), not all families cangeta chance to harvest the grass every year. The few families that do get a chance in a season can only harvest one bundle each, which is not enough to thatch one hut. Another interviewee from Matusadona National Park pointed out that benefits from CAMPFIRE are mainly collective, e.g., boreholes, and there are no benefits at household level. In the early beginning, CAMPFIRE benefits used to accrue at household level in form of dividends, but with population increases coupled with withdrawal of donor funding in CAMPFIRE projects, CAMPFIRE revenue has generally decreased and benefits are more generalised now. In terms of employment, one interviewee from Cawston Ranch mentioned that a greater percentage of all casual labour is sourced from the local communities. However, these kinds of jobs are seasonal and therefore not very dependable. With regards to permanent employment, another interviewee from Gonarezhou National Park pointed out that the park can only employ a few people of which only a small percentage comprises of local people and the rest are outsiders. Many of the employed local people occupy low positions with little income, e.g., lodge attendants and junior rangers. Moreover, many of local people lack the necessary qualifications to employ them in higher level positions. PA staff were aware of the fact that communities were not satisfied with the level of community employment in the PA. One respondent from Umfurudzi Park had this to say,

“Compared to the total number of employable local people, very few people benefit from employment in this park. Whilst we are trying our best, most the people do not seem to be satisfied. However, this is understandable, everyone wants a piece of the cake which can never be enough for everyone. But what can we do?”

Furthermore, according to one respondent from Gonarezhou National Park, the communities do not have a sustainable source of livelihood. Most of the communities rely on small-scale cash crop farming for income, which unfortunately do not give them much. Some parts surrounding Gonarezhou National Park are characterised by high temperatures and low rainfall, a climate which is not very conducive for crop farming. The respondent reiterated,

“This situation is worsened by wild animal destruction of crops as well as lack of financial resources for purchasing agricultural inputs”,

a problem which was found to be common in all the four study areas. One interviewee from Umfurudzi Park had this to say:

“There is limited capacity within the communities in terms of farming inputs which restrict them from realising better socio-economic benefits from crop production. Cash crop farming alone is thus not a very viable livelihood option for the communities hence the need for heavy reliance on wildlife resources.”

Besides, expectations from benefit-sharing which were partially met, other expectations were not met, for example, expectations for human-wildlife conflict and compensation for losses from wildlife. Expectations that were met had positive influence on PA-community relationships while those that were not met had negative influence (Table 5). Most PA staff in Umfurudzi Park and Gonarezhou National Park perceived their relationship with the community to be negative, while most of the staff in Matusadona National Park and Cawston Ranch PA staff perceived their relationships with the communities to be positive (Table 5). All communities(except a few community leaders with positive perceptions) reported negative perceptions on their relationship with PA staff whereas in two of the PAs (Umfurudzi Park and Gonarezhou National Park) PA staff perceived their relationship with the community to be negative, and in the other two (Matusadona National Park and Cawston Ranch), PA staff perceived a positive relationship with the community (Table 6).

**Table 6:** Summary of PA staff-community perceptions of their relationship.

Study site	Community						PA staff	
	Community leaders			Male heads	Females with families	Youths		
	`Majority view	Minority view	Unanimous view	Unanimous view	Unanimous view	Unanimous view	Majority view	Minority view
Umfurudzi			-	-	-	-	-	+
Gonarezhou	-	+		-	-	-	-	+
Matusadona	-	+		-	-	-	+	-
Cawston Ranch	-	+		-	-	-	+	-

Notes:— = negative; + = positive.

## Discussion

Benefit-sharing is a determinant of PA-community relationships that emerged from both communities’ and PA staff’s perceptions. While communities do getsome benefits, most respondents were not satisfied with the benefits, partly due to unmet high expectations and livelihoods that are strongly dependent on natural resources, in an environment characterised by a growing human population chasing dwindling wildlife resources. Local human population around PAs was further increased by the resettlement programme instituted by the government during the 2000 Fast Track Land Reform Programme in Zimbabwe, for example Yongwe, Kushinga and Sangoramambo villages adjacent Umfurudzi Park. As such, many of these community members may not even be from the respective areas and may dilute the benefit-sharing that could most probably go to groups who are long standing in the area. While quantifying the financial benefits from CAMPFIRE is complicated by factors such as the size of the programme and the increasing populations within the communities, the gross financial benefits among communities are generally very low [51]. Compared with the benefits obtained from agricultural production, the income from wildlife in most communities is purely supplementary although there are occasional substantial financial benefits, sometimes exceeding the estimated gross income from all agricultural sources [51].

Because the communities have many expectations, CAMPFIRE is overburdened by responsibilities, to the extent that proceeds from CAMPFIRE do not seem to satisfy everyone. Differences between minority community leaders’ views and the rest of the groups on CAMPFIRE benefits and other natural resources could be attributed to marginalisation of minority groups due to the fact that some traditional leadership performed a key part in controlling use of local resources with local people ending up as passive recipients of revenue derived from wildlife which they now view as belonging to the Rural District Councils (RDCs) or government [52, 53].

Our results on the impact of benefit-sharing on PA-community relationships concur with ‘s study of four PAs and their adjacent communities in Costa Rica which showed a link between the benefits communities receive and the perceived strength of the relationship between those communities and the respective PAs. The PA staff and community in each of the four study sites had similar views on benefit-sharing. While Umfurudzi community was not getting any benefits from the PA, Gonarezhou, Matusadona and Cawston Ranch communities received some benefits from the PAs as also confirmed by the PA staff. Our results from Umfurudzi support studies byand which showed that denied access to PA resources like grazing lands was a major cause for negative attitudes towards PAs in Ethiopia and South West Cameroon. Communities receiving few direct benefits tend to have negative attitudes as was the case in Gonarezhou. This concurs with previous studies conducted in Laikipia, Kenya and Western Serengeti, Tanzania, which reported that communities that receive few benefits than expected express dissatisfaction[27, 31].

However, it is also prudent to note that some of the communities’ expectations are misdirected at PAs. Moreover, some of the communities’ grievances such as not being able to illegally graze livestock in the PAs are outside the purpose of existence of many of the PAs. For example, according to, the purpose, significance and values for the park are to ‘protect and conserve the wilderness, biodiversity, ecological processes, wild and scenic landscapes within the park boundary. The park’s exceptional resource values will be sustained for present and future generations, while supporting its role in the Great Limpopo Transfrontier Conservation Area and regional economic development. The culture and history of the Shangaan people will be recognised as one of the key components of the park’. However, PAscan voluntarily assist by providing feeding schemes for animals outside the park, especially on a moral and ethical basis. While PA-staff have some expectations from communities like desisting from illegal hunting of wild animals, encroachment and veld fires, this does not influence any benefit-sharing schemes in place. Supporting wildlife conservation in adjacent PAs helps to promote wildlife tourism which can create business opportunities for adjacent local communities such as curio selling, accommodation and food outlets for visitors.

Human-wildlife conflict is a determinant of PA-community relationships that emerged from both communities’ and PA staff’s perceptions. The PA staff and communitiesin all the four study areas had similar views on human-wildlife conflict. All the four communities experienced some costs from wildlife in varying degrees. Human-wildlife conflict is one of the main threats to biodiversity conservation and has become frequent and severe in developing countries, especially in Africa[9, 28]. In Zimbabwe, the situation with human-wildlife costs is worsened by the fact that the Government is yet to develop a national policy on compensation for community losses due to wildlife depredation. However, elsewhere, compensation schemes of such a nature are at the time controversial[54]. For instance, proposes that it is better to address causes of the human-wildlife conflicts rather than address the symptoms because compensation can lead to a decrease in efforts to prevent damage and exacerbate conflicts with wildlife authorities.

Communication between PA staff and communities is another determinant of PA-community relationships that emerged from both communities’ and PA staff’s perceptions. A study by showed that improvements in communication was associated with an increase in the odds of having positive PA staff-community relationships in four PAs and their adjacent communities in Zimbabwe. PA staff in Umfurudzi, Gonarezhou and Matusadona reported that their communication with adjacent communities was open but limited. It was only in Cawston Ranch where PA management had scheduled meetings with community leaders. Similarly, all communities reported that communication wasinformal, irregular and insufficient. It is likely that the negative relationship between PA staff and adjacent communities could partly be attributed to this irregular and insufficient communication. Ineffective communication between PA authorities and local people can lead to conflicts [55, 56]. This result reveals the importance for PA management to examine their existing communication structure and ensure that effective communication is maintained. This could be done through increasing the frequency and the channels of communication, for example, by employing community liaison officers.

Community participation in the management of CAMPFIRE and/or tourism is another determinant of PA-community relationships that emerged from both communities’ and PA staff’s perceptions. The PA staff and community had similar views on community participation in the management of CAMPFIRE and/or tourism in PAs across the study sites. Although suggest that effective participation improves relationships, increases trust, and reduces conflict, none of the study communities participated in collaborative management of tourism in adjacent PAs. In contrast, community members from Matusadona and Gonarezhouhad limited participation in collaborative management of CAMPFIRE. Their limited participation in CAMPFIRE management meant that community members had no power to influence decisions, especially those regarding revenue-sharing.

Community perceptions of PA staff are a determinant of PA-community relationships that emerged from communities’ viewpoints whereas problems caused by communities emerged from PA staff’s viewpoints. Umfurudzi, Gonarezhou and Cawston Ranch communities had negative perceptions of PA staffwhich can be attributed to clashes between the communities and PA staff especially where illegal hunting is involved. confirmed that a total of 940 illegal hunters and 1,509 illegal fish poachers were arrested between 2000 and 2010 in Gonarezhou National Park, Zimbabwe. This kind of behaviour by communities may also cause PA staff to have negative perceptions about the communities. Communities’ negative perceptions of PA staff in Umfurudzi Park and Gonarezhou National Park was also mainly due to unfavourable changes brought about by public-private joint management of the parks, for example, the erection of the boundary fences, while in Cawston Ranch it could be because of the private nature of the PA. The communities’ negative perceptions, for example, in Cawston Ranch, could explain their lack of knowledge or understanding of the privately owned or managed PAs. As assert, privately owned and managed PAs are multiplying throughout much of the world and yet little is known about them.

A few community leaders from all communities had positive perceptions of PA staffthat they related well with, which can be attributed to the fact that community leaders are not usually involved in unsustainable activities like poaching and so are always treated well by PA staff. Moreover, some individuals from the communities who are employed in the PAs may have got their employment through recommendations from their leaders hence they are nice to them as a way of showing gratitude. When the PA staff have something to communicate to the villagers, they usually go through their leaders who will in turn inform the rest of the villagers. While this method is unpopular with the rest of the villagers, it brings closer PA staff and community leaders.

In Umfurudzi, both PA staff and the community had negative perceptions of their relationship mostly attributed to the lack of community benefits from the PA. This was largely due to the absence of any CBNRM project in Umfurudzi. Matusadona National Park staff perceived a positive relationship with the community likely because the community was benefiting in terms of employment and from CAMPFIRE. However, majority of Matusadona community members perceived a negative relationship with PA staff because as much as they benefited from employment and CAMPFIRE, the amount of benefits was perceived to be progressively declining over the years. This difference in perception between PA staff and the community presents a complex situation. To the PA staff, their positive perception could mean reduced pressure in terms of illegal hunting control efforts, whereas in actual fact illegal hunting is on the increase which has resulted in the rapid decline of elephants and other species in the park.

Gonarezhou community was benefiting from their neighbouring PA, for example, through employment and permitted access to park resources like thatching grass, but because there were often clashes between the PA staff and the communities due to illegal hunting, both PA staff and majority of the adjacent community members perceived a negative relationship. Contrastingly, Cawston Ranch community was benefiting from their neighbouring PA and because of this, PA staff perceived a positive relationship with the communities. However, the majority of Cawston Ranch community members perceived a negative relationship with the adjacent PA. This was mainly due to clashes between PA staff and the community because of illegal hunting. Moreover, in Cawston Ranch, the community was not happy with benefit-sharing structure where community leaders were perceived to be getting preferential treatment from PA management than the rest of the community members. This indicates a direct relationship between expectations and PA-community relationships.

Negative PA-community relationships have the potential to reduce local support for wildlife conservation [57, 58] who can, instead engage in activities that are detrimental to conservation such as illegal hunting and habitat encroachment [59]. The communities’ negative perceptions of their relationship with PA staff could mean that conservation problems like illegal hunting and habitat encroachment remain a challenge. However, PA staff’s positive perceptions about their relationship with local communities in Matusadona and Cawston Ranch is encouraging as lessons on positives can be taken and used in other areas with negatives.

We recognise that a division of PA staff, e.g., managers, senior rangers and junior rangers, would be very helpful given that they are likely to have some differences in perspectives. Thus we suggest future studies should consider such kind of division to capture more detailed information. While our results might be generally applicable to other PAs and their adjacent communities especially in developing countries, some of the issues raised are context specific (such as distribution of proceeds from CAMPFIRE or the effects of the erection of boundary fences on the adjacent communities) making the generalisability of this work limited. Furthermore, while the study assesses PA-community relationships from the views of both PA staff and local communities, we acknowledge that in some instances it may not entirely capture the complexities of how and why local people may behave towards PAs [60], as there could be exogenous factors influencing relationships external to the immediate parties involved.

## Conclusion and recommendations

Seven determinants of PA-community relationships emerged from both communities and PA staff’ expectations. While majority of community members in all the four communities reported negative perceptions on their relationship with PA staff, PA staff perceptions of their relationship with local communities varied from negative to positive. Both communities’ and PA staff’s views on determinants are almost similar in all studied PAs regardless of PA ownership, management or land use. We conclude that although investigating communities’ expectations is important for building and maintaining positive PA-community relationships, it is important to understand that what communities expect may often be different from reality. In most cases communities’ expectations are misdirected at PAs instead of the proper responsible authorities. As such, communities may always be dissatisfied with PAs and this may undermine PA efforts to build and maintain positive relationship with adjacent communities. It is therefore important to educate communities about different entities and their responsibilities, including PAs, CBNRM projects, Local Government and Non-Governmental Organisations. Educating communities on how to properly communicate and channel their grievances to the responsible authorities is also important.

Although initiatives like CAMPFIRE may contribute to positive relationships between PAs and adjacent communities, our findings suggest that such initiatives alone are not enough to guarantee positive PA-community relationships. Other determinants like communication and human-wildlife conflicts also need to be carefully considered. Our results can be used by policy makers especially in developing countries to develop national PA-community relationship frameworks based on these findings. A PA-community relationship framework represents factors that influence relationships between PA staff and adjacent communities which can be used to shape PA management strategies to both PA staff and local communities’ attitudes[56]. The framework offers a systematic way to conceptualise the factors that both PA staff and local communities need to address in order to promote positive PA-community relationships. PAs would benefit from the use of the framework to address factors that influence PA staff and local community relationships, and pressures on resources at different levels. Furthermore, PA agencies and adjacent communities should continuously seek to improve collaborationbetweenbothparties, and address all the determinants which help improve their relationships.

## Supporting information

S1 Table Focus group discussions data. (DOCX)

S2 Table Interview data. (DOCX)
