# Correction: Protected area staff and local community viewpoints: A qualitative assessment of conservation relationships in Zimbabwe

**DOI:** 10.1371/journal.pone.0184779

**Published:** 2017-09-19

**Authors:** Chiedza Ngonidzashe Mutanga, Never Muboko, Edson Gandiwa

There are several references which were omitted from the article. The first sentence of the second paragraph in “Community perceptions of determinants and PA staff-community relationship” in the Data collection section should read: Ten discussants were targeted for each FGD (Table 2) as recommended by Krueger and Casey (2000).

The reference is: Krueger RA, Casey MA. Focus groups. A practical guide for applied research. 2000;3.

The first sentence of the fourth paragraph in the same section should read: Table 2 shows the distribution of community members who participated in FGDs and the estimated population of community members in a district (population statistics were derived from Zimbabwe National Statistics Agency (2012)).

The reference is: Zimbabwe National Statistics Agency. The Zimbabwe 2012 Population Census National Report Harare: ZimStats; 2012.

The first sentence in the Data analysis section should read: Following Ormsby and Kaplin [56], qualitative data from both FGDs and interviews were analysed using content analysis where the key issues were grouped into themes.

The first sentence of the third paragraph in the Discussion should read: Our results on the impact of benefit-sharing on PA-community relationships concur with Molina-Murillo et al.’s study [39] of four PAs and their adjacent communities in Costa Rica which showed a link between the benefits communities receive and the perceived strength of the relationship between those communities and the respective PAs.

The fourth sentence in the same paragraph should read: Our results from Umfurudzi support studies by Tessema et al. [26] and Ebua et al. (2011) which showed that denied access to PA resources like grazing lands was a major cause for negative attitudes towards PAs in Ethiopia and South West Cameroon.

The reference is: Ebua VB, Agwafo TE, Fonkwo SN. Attitudes and perceptions as threats to wildlife conservation in the Bakossi area, South West Cameroon. International Journal of Biodiversity and Conservation. 2011;3(12):631–6.

The third sentence of the fourth paragraph in the Discussion should read: For example, according to Gonarezhou National Park (2011–2021), the purpose, significance and values for the park are to ‘protect and conserve the wilderness, biodiversity, ecological processes, wild and scenic landscapes within the park boundary.

The reference is: Gonarezhou National Park. Gonarezhou National Park General Management Plan Harare, Zimbabwe: ZPWMA 2011–2021.

The seventh sentence in the fifth paragraph in the Discussion should read: For instance, Bulte and Rondeau (2005) proposes that it is better to address causes of the human-wildlife conflicts rather than address the symptoms because compensation can lead to a decrease in efforts to prevent damage and exacerbate conflicts with wildlife authorities.

The reference is: Bulte EH, Rondeau D. Research And Management Viewpoint: Why Compensating Wildlife Damages May Be Bad For Conservation. Journal Of Wildlife Management 2005;69(1):14–9.

The second sentence in the sixth paragraph of the Discussion should read: A study by Mutanga et al. [42] showed that improvements in communication was associated with an increase in the odds of having positive PA staff-community relationships in four PAs and their adjacent communities in Zimbabwe.

The third sentence in the seventh paragraph of the Discussion should read: Although Beierle and Konisky (2001) suggest that effective participation improves relationships, increases trust, and reduces conflict, none of the study communities participated in collaborative management of tourism in adjacent PAs.

The reference is: Beierle TC, Konisky DM. What are we gaining from stakeholder involvement? Observations from environmental planning in the Great Lakes. Environmental Planning C: Government Policy. 2001;19:515–27.

The third sentence in the eighth paragraph of the Discussion should read: Gandiwa et al. [59] confirmed that a total of 940 illegal hunters and 1,509 illegal fish poachers were arrested between 2000 and 2010 in Gonarezhou National Park, Zimbabwe.

The seventh sentence in the same paragraph should read: As Langholz and Lassoie (2001) assert, privately owned and managed PAs are multiplying throughout much of the world and yet little is known about them.

The reference is: Langholz JA, Lassoie JP. Perils and Promise of Privately Owned Protected Areas. BioScience. 2001;51(12).

Reference 42 is incomplete. The reference should read: Mutanga CN, Muboko N, Gandiwa E, Vengesayi S. Beyond a single perspective to conservation relationships: exploring factors influencing protected area staff and local community relationships in Zimbabwe. International Journal of Biodiversity Science, Ecosystem Services & Management. 2016;12(3):212–26.
