# Supplementary material for: Protected area staff and local community viewpoints: A qualitative assessment of conservation relationships in Zimbabwe
Source: PLoS One. 2017 May 19;12(5):e0177153. doi: 10.1371/journal.pone.0177153 (PMC5438145; doi:10.1371/journal.pone.0177153)
Supplement: S1 Table — (DOCX) [file pone.0177153.s001.docx]

**S1Table: Focus Group Discussions (FGDs) Data from Four Communities around Four Protected Areas (PAs) Transcribed Verbatim**

| **PA 1**  **FGD 1**  Our relationship with the park is very bad. Imagine I came here in 1980. The park has never helped us. Up to now, I don’t think the park has helped us in any way.  There is no relationship to talk about; graves of our ancestors are in there. Our ancestors were forcefully removed from that place by the white colonialists; all that area belongs to Chief Nyamaropa.  Before park was created our ancestors used to live in that area and the graves of our forefathers are still there in the park. The coming of park resulted in our fathers being pushed out of the park, leaving behind those grave yards. So according to our tradition we need to have regular visit to those shrines to appease our forefathers or just to clean their grave yards. This is not happening easily as the park is not allowing us, they asked to apply for permit to enter and carry out those ceremonies, a situation which is not fair at all  These people are just staying in my area, and are not doing anything for me. The tourists come and pay but we do not know where all that money goes to, because we, the people, are not getting anything.  They don’t want to involve us in their tourism. We hear it is very good business and the tourists come with lots and lots of money, but because they do not want us to develop they make sure we are kept in the dark.  These people do not even respect our leaderships and they don’t know the importance of communication. Can you imagine they did not even tell the king that they want to erect the fence in our land. The do not respect our leaders.  The park staff do not relate well with us. They are cruel and mean. You see as old as I am now, they beat us with no shame at all.    Here we do not kill wild animals, if we see them destroying our crops or livestock we call the parks people to come and get their animals. So you see, we actually want to live well with them but it is them who do not want a good relationship with us.  We are not happy with the park because their animals destroy our crops and eat our livestock and we have never received compensation of any kind.  Before the recent coming of the white people who are managing the park now, our situation was better because the animals were very few. Now they are re-introducing the animals which are always roaming around, we are suffering from a lot of destruction of our crops and livestock, and yet we benefit nothing. In a nutshell, this park is causing us more harm than good.  We have celebrations that we hold every year for example the Independence Day celebrations and the Heroes Day celebrations. We always go to them to ask for game meat, but each year it is the same story, “You are late.” The following year we make an effort to go early enough, and then they say “we are processing it”, until it past the day of the celebrations. So you see these people do not care a bit about us.  If only parks could push their fence inwards so that our livestock can have somewhere to graze.    Actually people, the park is important, it has to be there. But it has to benefit us the neighbours.  It is our right to benefit from the park but we are not getting anything  Our cattle are dying of hunger. Through fencing, the park has denied us access to our grazing land. They are oppressing us.    If we ask for anything we can wait from six months to a year without getting a reply. This can go on and on until we just keep quiet about it.  We have not yet received any benefits from the park.  We do not have a good relationship with the park.  If we ask for help they ignore us, they do not care about us.  We do not support people who poach. They are criminals and they should be punished. But all of this is the park’s fault; it is because of them that people are suffering.  Just last week our son was beaten up and arrested because they accused him of killing a giraffe. When our elders went there to understand what had happened, they were beaten up arrested too.  **FGD 2**  We have the worst relationship ever with the park. This is our land and these are our animals. Under normal circumstances we should be able to do what we want with our animals, if we want meat we should be able to kill the animals like our ancestors used to do long back. But now if we do that we are arrested, that is why we do not want thus park here. It is better if they take their park away from our land.  The park and us, we don’t get along well. Before the joint venture, and the coming of these white people, we could negotiate with the park so that we could mine and pay our fees later but now, these white people do not allow us to do that anymore. We do not have any other source of income and our children are dying of hunger.  We want to take care and visit our ancestors’ graves but they do not let us. Actually, we are not getting anything from this park, and as a result there is nothing we admire about the park. If at all it is possible, they should just remove it from here and let us stay in peace.  They should build some dams for us so that our cattle will not die of thirst just like they build dams for their animals. At least they owe us that much considering that we are not getting anything from the park.  We hear about this thing called CAMPFIRE, but we do not have it here. As such the park does not benefit us in any way. If the park would at least, build us schools, roads, dams and help us with electricity we would be very grateful.  They brought their cheetahs here. Now four of my cattle were killed. As if that is not enough, our crops, especially those of us who are close to the boundary, are always destroyed by the kudus and sables. The worst part is that up to today, I have not been given even a single cent for my losses  We lose a lot of our livestock to their animals but no one has ever been compensated.  I have stopped mining in the park for close to five years now because of the exorbitant fees they charge us. I have a mine near the Six area, and i have two other mines further down. I have since closed all of them down because I can’t afford the high fees they are charging. Where can I get an amount close to US$10,000 which they are demanding?  This park is useless to us; it does not benefit us in any way. For example, we have a primary school close by, our kids there do not even know an elephant, those who have an idea have seen it in books, but we live close to the park. What can be more insulting than that? If the park wanted to be fair at least they should extent some services to us for free like free entrance into the park for the local children on school trips.  One of the reasons why we keep cattle and goats is so that we can sell them whenever we are in need of cash for say school fees for our children or fertilisers. So if our livestock are killed, how are we going to survive then?  They are slow in erecting their fence. As a result, their animals come in groups to destroy our crops. Those buffalo herds are really scary.  Now we are off the farming season, if we release our cattle they go straight into the park because that’s where the grass is. They hold our cattle, and if we go there to collect them they hold us too. Who does that to their neighbour?  Why should we be overcrowded here when there is too much land in the park? At least they should give us some more space to build our houses. Our sons need to build their homes too.  They make us pay fines for our stray cattle they hold in the park  Sometimes the buffalos come out of the park to graze in our land. A few weeks ago we lost a number of cattle that grazed where the buffalos had grazed.  Our mines are being taken by people from Harare because they have got the money. We are left to suffer because we have no money. The park does not care about is at all.  We love the park but we need to have an understanding so that we can live peacefully. If we need something they should at least hear us out. That way we can also hear them out when they need something from us. For example they cannot expect us to protect their animals when they do not care about us. Now if we see their animals, we will not protect them, what will happen, will happen.  They underestimate the power of communication and they do not even value it. That is why they never communicate with us or our leaders about anything.  When they erected their fence, they took parts of our fields.    We do not have a buffer zone. If their animals come out, they are in our yards. The park should move their boundary inwards and at least leave a buffer zone.  Our children want to go to school but they are at home, and yet we live close to the park. Isn’t that an irony? The park should help us build a school, clinic and many other things. That way we will know we are living closer to the park.  As big as our area is, we only have two boreholes. People travel for long distances to fetch water and the park is not doing anything to help solve the problem.  Many of our children do not get employment from the park as the park only employs very few. Moreover, we can no longer mine in the park because we cannot afford the fees that they ask for. That is why we kill and sell their animals, we only want to survive.  If our cattle get inside the park, they hold them until we pay the fines they demand from us but their animals come out here too and destroy our crops do not pay us anything? How unfair this is?  We used to get water from the park but now with the fence it is not possible anymore. Since they decided to put the fence we thought they could at least be human enough to drill a borehole for us.  Since the Fast Track Land Reform of 2000, many people have come to live here and those are the ones with land encroaching into the park and this has only increased our problems with the park.  The park is playing a hide and seek game with us. Why is it that they do not come out in the open and tell us exactly where we stand with the park, how are we supposed to benefit and so forth?  We are not allowed to enter the park if we have no money which means we are never going to enter the park because we have no means of getting the money.  The park is good for the conservation of wild animals and plants. The plants are good for oxygen, so although the park is not benefiting us materially, we are happy to live close to the park. At least if they would give us an opportunity to learn about tourism and also benefit from it, then we will both be happy, the park is happy and we are happy.  Many people who work in the park come from Masvingo, Bulawayo and Mutare. Very few of our children from here are employed in the park. Where are the rest of children supposed to work or does it mean are children are so dump that cannot even cut grass or construct a fireguard?  The white person who manages the park does not even want to see us, actually, he says we are baboons, so how are we ever going to relate with him? He has actually passed this hatred to all the workers in the park; they do not even greet us when we meet on the road. All they are good at is to victimise us and treat us like poachers.  You know, the only reason why this park is still here is because of the respect we have for our Chief who continuously asks us to take it easy, otherwise we would have chased them away long back.  We have lost so much livestock since we started living here about 33 years ago. My neighbour here recently lost four cattle which were attacked at night.  We lose livestock to animals like cheetahs. The woman two houses down the line lost two goats. Elephants invaded the same woman’s garden and destroyed everything including cabbages which were due for market.  The wild animals spread diseases to our livestock especially the buffalo. Because we cannot afford the medicine that is required to treat them, we eventually lose many of our cattle due to illnesses.  At least they should pay us for all the livestock and crops we lose to depredation by their animals.  People who come from outside are the ones that are allowed to pay money and hunt. Why can’t they ask for small fees from us too, say US$100-00 so that we are also able to hunt? What it means here is that tourists are more important to them than us, as a result tourists are the ones who are benefiting from the park, not us.  The park should help us build schools, good roads and even help with electricity.  The park came here to oppress us. We would be better off if they left.  We are losing so many goats to hyenas. The hyenas are really bad news to us.  We have a very bad relationship with the park.  Those of us who share the boundary with the park suffer most in terms of crop losses to kudus, sables and warthogs year after year but the park does nothing to help us.  **FGD 3**  Our relationship with the park is bad. The animals are dangerous especially the elephants. We are even afraid to walk around when we see them.  How can we have a good relationship if the park staff are very rude and uncaring? All they can say is that if you kill such and such an animal you pay so much or you go to prison, but every day we are losing our livestock here, who should we report to? Who should pay us? They want us to pay for their kudus but they can’t pay us for our cattle. This is not a fair game.  Here most of us are very poor and health facilities are located very from our homesteads, some are as far as 30 kilometres away. As women in most cases we need to go to those clinics to seek medical attention, like giving birth or just seeking medication, when we or our children and even husbands are not feeling well. Whenever you meet the park vehicle on our way to clinic or any, in the direction in which we will be going, they never offer us a lift unless we pay them or else they leave us behind.  We are true Zimbabweans, we support the park. From the newspapers we read, we learn that other countries no longer have wild animals. Many tourists who come here come to see these animals, which is good. But the problem now is the park takes all the money that the tourists bring and the community gets nothing. Right now there is little public transport on our roads and many times we travel for several kilometres before we can get any transport but the park just turns a blind eye.  The parks should learn to help its neighbours, that way; we can live together in peace. Actually, a good starting point would be working on their communication skills. We know that if you go to other people’s homes it is disrespectful to just do what you want without communicating with them. That’s exactly what the park does here. They don’t communicate at all. We just wake up one day and we see people who are busy working on the fence, what?  The park which is the government arm does not even come to people to teach us about the reasons for the existence of the park and how it should benefit the people. As people who live closer to the park, we cannot say we know much about the park, but all we know is that the park is not benefiting us in any way. If they involve us in tourism, we could all benefit you know.  The animals only benefit people who come from America. How is this supposed to help us then?  Since the park cannot compensate us for our losses, they should find ways of keeping their animals inside so that they do not disturb us.  The park is good. Now we know different kinds of animals like the giraffe, buffalo and elephant.  Many jobs arise in the park but to our surprise a few of our children are absorbed and the rest are foreigners. Why?  We want to learn about the park as we do not have much knowledge on its operations but the park does not even give us the opportunity to do so.  Tourists are the reason why the park treats us so badly because they bring money and we don’t. So we do not care about their tourists, whether they come or not we are not bothered, whether they are happy or not we do not even care.  **FGD 4**  We have a very bad relationship with the park. We are just slaves to the park; we are not involved in anything they do there. All they know is to treat us like, thieves, poachers who do not know how to do anything else. They harass us all the time, they accuse us of poaching. Those rangers are wicked, I don’t even think they were trained for that job, at least they should know how to investigate before torturing people for things they did not do.  Look, we are not saying there are no poachers, and we do not condone poaching either. If those people are caught, it’s true they should be punished. All we are saying here is that not all of us are poachers and so not all of us should be treated as such either. These rangers should at least try to be polite. Maybe that can improve our relationship a bit, just maybe.  They do not communicate well with us. All they do is dictate to us and give us rules about we should not do, otherwise we will be arrested. We never have a two-way communication, we are suppressed always. This is one reason why our relationship is bad.  One time the park staff brought us pamphlets with stipulating the law with regards to killing of wild animals, especially the fees that we have to pay for each and every animal species that we kill. What was surprising was that it mentioned nothing about what the park should pay people who lose crops, livestock, property, even lives or at least how it should help people.  We know animals are important but you want to know why we poach. The park does not want to employ us but we also want to make money. There is nothing else we can do here, we want to start projects but we do not have the start up capital, if only the park could help us start some project that can help us raise some money.  Some of us did not pass well our ordinary level exams, some did not even finish school due to lack of school fees so we cannot go to the towns to look for employment. Which company will employ us there? Regardless, we still need to survive, so we have to do what we can here.  We also need some stable sources of income, one day we are going to get married and we will have families to care of, so you think we are happy about this our situation. We know that what we do at times is not right and not sustainable but believe me, we will be desperate. You know what’s worse; the park does not even want us to be involved in tourism. They should at least give us a chance, train us, or at least hear us out. We can work something out together and find ways in which we can be involved in tourism and benefit from it too.  It’s all the park’s fault. They are supposed to help us but no, they enjoy all the benefits themselves.  We try to grow crops but because we live very close to the park boundary, every year we lose many of our crops to animals, what we are usually left with is hardly enough to survive on let alone sell to get money. The park does not even give us anything to pay for our losses; we are tired of going to register what we lose at the park. Every time they say, “we will see what we can do”, but the truth is they have never bothered to see to anything. |
| --- |
| **PA 2**  **FGD 1**  Relationship? That’s a very sour and painful topic. For starters, we are made to pay fines if our cattle go into the park. Although we like the idea that animals are being conserved, we are not happy with the way the park handles their affairs. We don’t charge them if their animals destroy our fields, livestock or property. Actually, we would love to but we can’t. So you see it is a one way relationship, why can’t we do to them what they do to us. The park staff treat us really bad.  We do not even have such a thing called relationship. Our relationship with the park is like trying to mix oil and water. It can’t be possible now, can it? The hyenas are eating our cattle. We tell park staff but they do not do anything about it, so what do you expect?  You know this new project they have in there? They call is Conservation project which is being run by the whites. That project brought unfavourable changes to us. It is them that introduced the fence now; even CAMPFIRE cannot be viable under such circumstances, not to talk about our cattle.  The park should give us a small portion for our cattle to graze. Also they should increase the number of locals they employ.  The park does not involve us in anything they do even if they know their decisions will affect us. At least they should notify us well in advance so that we prepare ourselves not to just surprise us with bombshells like that. When we grew up, we were told communication is very important, has that changed now? Because it looks like these people from the park do not even know the meaning of that word.  No, of course the park can be bad at times yes, but we cannot rule it out completely. It can’t be that bad, here and there we relate quite well. There are a number of things that we are grateful for. For example, CAMPFIRE helps us a lot. Besides communal benefits like grinding mills, hardware store, truck and tractor, people enjoy individual benefits like meat from the hunted elephants and occasional cash dividends.  We know and we are grateful for being involved in CAMPFIRE management, but we still feel the park needs to involve us more especially in tourism within the park. Do you know that our people are not even allowed to sell their curios or beads to tourists in the park? Because of this, we will the park is ripping us off of potential opportunities to upgrade our lives. Our people have got potential I tell you.  The park does not consult us. They do what they want and we just at the receiving end. If they were considerate enough we would not be worrying about not being consultant but the problem is it looks like the park enjoys seeing us suffer and they will always make decisions that oppress us. The park staff is really rude.  The park is our enemy here.  Last year we buried a pregnant woman who was killed by an elephant.  If small elephants get out of the park we are doomed because the hunters won’t kill them because of their size, but regardless of how small they are, they will still destroy our crops. So in the end we lose both ways.  You know, these parks people really surprise me. They think they know it all when it comes to conserving animals forgetting that before the park was even here, we used to live in harmony with the animals and conserve them using our traditional ways.  **FGD 2**  The relationship between the park and us is really bad. To think that we are arrested if we are found in the park and yet all that land is ours. The park and the government it represents are all unfair. It looks likes these rangers have got some course of cruelty that have to undertake and pass before they are employed. I tell you I have never seen anyone so harsh and rude all my life.  We have problems getting pastures for our cattle. As we speak now many people have migrated to Mozambique. My children and cattle are in Mozambique right now. So maybe you can work out how bad our relationship is, it’s in black and white, isn’t it?  They fenced the park, now we no longer have grazing land or water for our cattle. Our cattle are dying in numbers.  Something is strange though, you know this fence has not stopped animals to come out puff the park to harm us, to destroy our crops and to kill our livestock. Its purpose is to prevent our cattle from going in there. We are still losing our crops and livestock, well, maybe a little less than before, but we still are, and still there is no compensation for our losses. We kind of feel as if the blow is harder now we have to lose our cattle due to hunger too.  Putting the fence was the cruellest thing the park has ever done to us. Our cattle used to be our source of income. If you had cattle you would not die of hunger, your children would not be sent back from school, now that the cattle are all dying, people are also dying of hunger and children do not go to school any more. Look, you see how thin those cattle are, they are just moving bones. Even if we were to sell them for as little as a $5-00 each, no butcher would buy them, they say the meat is no longer suitable for human consumption.  This new conservation project with whites started last year. Before that we were fine here but this project brought the fence and our cattle have nowhere to graze. Since the project started last year we have lost more than 500 cattle due to hunger.  So far I have lost 27 cattle and my children no longer go to school. The park does not seem to care; I do not know what to do anymore.  We have never benefited anything from the park. The shed that is used for settling disputes, which was constructed this year, is the only thing that the park has done for our community. By building us that shed they thought they were deceiving us, they must think we are very stupid.  It is good to conserve wild animals but this should not disturb our life.  Our children often get employed in the park but rarely.  If we have issues we try to communicate and reason with the park but this has never helped before. The park itself doesn’t want to hear us out, they don’t want to engage us, they won’t talk us, so what can we do?  The park does not even want to see us here; they are always finding reasons to fight us. I think if it was really up to them we would all be dead now.  This area does not benefit much from CAMPFIRE. CAMPFIRE benefits more in those areas where the Member of Parliament (MP) comes from.  The park does not communicate with us; they do not even hold meetings with us.  You know what, even we do not support people who poach, if we have animals that come out of the park, that’s great, that is why CAMPFIRE is there and that is how we benefit also.  Park staff do not treat us well, they arrest people based on mere suspicion. In most of the cases they do not have evidence to back their actions.  We are hurt because tourists visit this park a lot, and yet we do not benefit anything from tourism. There are of course a few who are involved in the traditional hut project and who are benefiting from it. But we feel we can be involved a bit more, that way, all of us can benefit.  We benefit a lot from CAMPFIRE. We have a truck and a tractor that were bought with money from CAMPFIRE and which help people from the community if they want to carry personal loads like bricks, river sand, pit sand and so forth. We also have grinding mills and a hardware store that were brought by revenue from CAMPFIRE. People also benefit in form of cash dividends per household and meat from culled animals.  Some people go to poach animals in the park. Later on the park rangers may discover footsteps and decide to follow the poachers in the villages. More often than not the beat and arrest the wrong people.  Park staff are very cruel, they act on rumours, for example, if they hear that someone has got dogs, they automatically assume they use them for poaching and will come and beat up the whole family from children to women to grand fathers and grand mothers.  What park staff do is not right. How can you just beat up people without enough proof? If the real police were to operate that way, by now the whole country would have collapsed. It is not human.  **FGD 3**  Our relationship with the park can never be good. The Park erected the fence without even consulting us, our children used to go to school because we would sell the cattle to get money for school fees, but now they no longer go to school. In times of hunger, we would sell the cattle and use the money to buy food. Now because of this fence, our cattle are dying and those that are still alive are so thin that nobody wants to buy them. How then are we supposed to live?  Of course the relationship is very bad. When they erected this fence, they did not consult the people. Our cattle are dying of hunger and so are we.  The fence has reduced revenue from CAMPFIRE. Before the fence was there, elephants used to come out of the park in numbers and many of them would be sold to hunters and we would get meat and of course the money for projects. Now with the fence at times we get very few elephants.  We are really suffering. Our cattle used to survive on pasture from the park but now they are starving to death.  For our cattle to survive we have to take them to Mozambique where we asked for pasture for a specified period of time in exchange for a fee. For example, to graze ten cattle for six months, we pay one cow. If we want to renew the contract after six months we pay another cow.  Here we survive because of cattle. Now that we do not have cattle, we are all going to die if the government does not intervene.  Some people cut some parts of the fence sometime ago. In reacting to this tragedy, the park called for a meeting (something which they almost never do by the way) and they said, “You are not allowed to cut the fence or anything that belongs to the government. What you can do is to sell all your cattle whilst they are still sellable and look for other projects to do with the money because if you don’t, all your cattle are going to die of hunger. The park cannot help you with this one”. So tell me, if we sell all our cattle how are we going to survive, how are our children going to go to school? In any case what kind of communication is that? Is that even normal? This fence thing certainly worsened our relationship with the park. Also worrying is the harshness portrayed by the park staff. Any normal human being should at least have some level of compassion, but these park people, they were cut from their own cloth.  The thatching grass that we are allocated at any given time is not enough to roof one hut. So if you get this insufficient portion this year, you would have to wait for another year to get another portion. It’s not practical at all, how can you roof a house like that?  In our ward, ward 5, there is not even a single person who is employed in the park.  We will always be lagging behind in terms of development. Imagine that our children are no longer going to school because we no longer have cattle. Surely our children no longer have a brighter future.  Before the erection of the fence, CAMPFIRE used to realise more revenue, and we also used to benefit from that, but now the animals that cross over are reduced in numbers and so are the benefits.  We know of the existence of the park and we hear about tourists. The truth is we have no detail or knowledge about the tourists or tourism. Nobody has ever cared to educate us about tourism and how we can befit from it.  The actual boundary between the park us is further down there not nearby where they put it. They just put the fence here so that our cattle would die of hunger. These people are heartless you know.  The presence of the fence is good on the other hand because now the hyenas and the elephants do not destroy our crops and livestock anymore.  The park does not communicate with us well. Right now if we have issues we do not know how we can communicate with the park.  We have no CAMPFIRE in our ward, which is okay but we do not get any benefits at all from the park. Even this CAMPFIRE, we are not even happy with the way it is run. Why are we not involved? The District Council together with our leaders who are certainly benefitting more from this CAMPFIRE will always do what they want anyway.  We are just in the dark. We do not receive any education about the tourists, what we always see are the tourists driving in and out of the park and that’s all.  If we have a function like the Independence or Heroes celebrations that we always have every year, we always ask for an animal for meat but they always refuse. One other time they even gave us money to buy a cow, what insult.  We are even afraid to speak out our minds for fear of getting arrested. These people are cruel.  We are Shangaan, we use cattle to survive. Every family had more than 50 cattle but now all of them are dying due to lack of pasture.  If this park was a person we would go to a witchdoctor to kill him because we are really suffering because of the park.  Instead of us to benefit through the park, we are actually suffering.  You know the purpose of this fence? It is so that our cattle do not go into the park and not the other way round because elephants can always come out and they still destroy our crops.  Before they put the fence we would thatch our school every year because we could get the thatching grass from the park, but now we no longer thatch our school and when the rain season comes our children do not go to school because we can’t afford zinc or asbestos roofing sheets.  When the park was first created our ancestors were forcefully moved out of their place that is why we took the opportunity to claim our territory back in the park in 2000 during the Fast Track Land Reform.  We enjoy a few benefits from the park for example thatching grass although we are limited.  We request for many things from the park, some of which are denied like game meat for our celebrations. However some requests are granted for example every year we are allowed to have our cultural ceremonies in the park which we hold to honour our ancestors.  The park people are very unpredictable. If we take our requests to the park, they sometimes grant what they can and forward to the head office those that they cannot’.  The park values animals more than us. Actually, they do not even want us here. We lose our crops and livestock and they do not care even a little. Fine we have since accepted the fact that they do not compensate us for our losses, but they should at least try to respond early if we call them about problem animals. Instead they give all sorts of excuses like shortage of transport or no fuel. But if they hear rumours about poaching, they don’t even take time to arrive. Surely, we can’t help wondering what really their issue is. Government should just give us transferring letters so that we relocate to Mozambique and join some of our people there.  Before this confusion that the fence has brought about we were almost living in harmony with the park.  **FGD 4**  What relationship are you talking about? We do not have a relationship with those people. Okay, let me start with this one: the thatching grass that we are allocated is not even close to enough and they do not allow us to gather firewood. How about that?  Actually, this new park management is terrible. We were better off when it was the Government managing the park alone. We don’t get along well at all.  Here and there we get thatching grass but it is not enough. We want to be free to get the grass whenever we want.  We love the park, we only wish the park staff could be a bit considerate especially with the way they treat us the youths. Instead of looking at us as if we were thieves all the time, why can’t they help us do something productive like teaching us some skills so that we begin some projects that can help earn money. All they do is come after us all the time with accusations of poaching and beating us up. Are they ever going to get tired?  We cannot sincerely say we benefit from CAMPFIRE. Probably yes, but are voices as the youths are not represented anyway. We want to be visible and we certainly want to be useful. We have got great ideas but we are never given the opportunity and we are not involved in anything.  If something is happening in the park, we hear about it through rumours. Can’t they at least have some formal and more organised way of communicating? We have respect for the park, at least they should treat us with a little bit of respect too.  We want to learn more about tourism so that if there is any way we can participate and benefit from it, then that would be great, but the park does not teach us anything.  We need employment. The park does not want to employ us and yet that should be one of our benefits. Of course we know they say they do not have the capacity to take many people but that’s too much, there is one family in the next village where the father is employed in the park, then we know of two more down there who are employed. We know all about it, the park is full of people from outside. Do they think we are stupid or what?  These people are cruel and we are now tired. They come into our homes to harass us and accuse us of poaching. Did you know that is even a crime to own a dog here? If you have one you are always harassed and beaten. Sometimes we think that these people do not have work to do so they want us to poach to that they will feel useful.  What we do is not poaching; we simply take what belongs to us. If we kill these animals they say we have committed a crime, the animals belong to the government, but if the animals destroy our crops and eat our livestock they say we cannot compensate you because they are your animals. This Government or the park should take a position, are these our animals or not? And if they are, so why can’t we share the money from tourism then? |
| **PA 3**  **FGD 1**  We can say our relationship is not good. This is because we still feel this bitterness that our grandfathers were forcefully removed from that area when the park was created. Okay, let’s just say what is done is done, but at least they should have put a fence to keep their animals there. Instead, they let them roam freely and they are destroying crops, livestock and even people.  The park used to listen to us probably because they closely worked with the chiefs and other leaders, but these days, we don’t work together anymore and they do not listen to us too. That is why people chase them with spears when they come here following up on suspected poachers. Our relationship is no longer good at all. The park staff are often rude to our people that is why there is always war between them.  The relationship between the park and us is neither bad nor good. Actually it is moderate; there are certain things that we like and certain things that we do not like. For example, although they often take long to respond to complains, PA staff are cheerful and they relate well with us. We drink beer together in beer halls and they even come to our homes for beer when they are free. They communicate with us well too. They give us their messages and everything they want the people to know and we pass the information on to the people. The same happens when the people want to communicate with the park, they come through us and we facilitate the communication.  We feel we are not benefitting much from the park. The park should give us a percentage of the revenue they earn from tourism so that we also benefit something.  The schools and clinics in this area are proceeds from CAMPFIRE. We have at least benefited something.  External poachers are frequenting this area a lot these days and because of them we hear that animal populations are getting low. If our people help these poachers it is because they are not happy, they have no other source of income, and so any way that brings in a little cash is always welcome. But come to think of it, we do not get revenue from CAMPFIRE anymore, and even if we did we do not get benefits at household level, we try farming, our crops are destroyed by animals and we don’t get a single cent for the losses, and the park cannot even absorb a tenth of the of our men and youths to work in there, so what can they do really?  Within our midst there are poachers too, some who kill animals for sale, others for subsistence, and the worst kind are those who collaborate with external poachers and these operate on a larger scale. They usually kill animals like elephants for the ivory. If you ask me, I say it is the park’s fault, the park expects us to help them, to work with them but they do not return the favour. Many tourists come in there but they do not give us an opportunity to sell our staff to them, since they can’t give us a share of the revenue from tourism, at least they should allow us to benefit directly from the tourists now.  We are in good books with the park. If we have a problem they help us if they can for example, during funerals, they often help us with meat.  Our children get jobs in the park and sometimes in other tourism companies, although they are not many who get this benefit.  **FGD 2**  Our relationship is not good. The main challenge we face is that our crops are destroyed by animals and we do not get any compensation at all.  The park does not do anything for us at all, we channel our requests to the park through our leaders but the park just turns a deaf ear, they do not anything at all.  We do not support poaching here. There are many poachers here who mainly come from Zambia and each time we see them we report them to the park.  Every year we lose our crops to wild animals. The park does not help us in any way even if we lose everything, they simply do not care.  The park staff do not care about us; all they care about is the welfare of their animals. As such they do not even want to see people in the park.  Parks staff are inhuman, whenever an individual is attacked by wild animal or when our crops are raided by their animals, they do not come to our rescue in time , but should it happen that they received a report of suspected poaching from one of the community member they react immediately even with limited resources. For example in 2014, we reported a situation were by crocodiles were preying on our goats, we lost about fifty goats in that year, we approached park officials to come and kill or trap the crocodiles but they never come, citing unnecessary reasons, such as manpower shortages and unavailability of vehicle to react. However what surprised us is that, one of the days, in that same period when we were losing our goats, one of the local people was suspected to have poached an impala, which has the same size as the goat. They reacted within few hours of receiving the report, and arrested him. So we realised that, what is important to them are wild animals not humans.  We get meat from culled animals so CAMPFIRE is good to us.  We hear that tourists come to the park in their numbers but we are not benefiting anything from tourism. The park won’t let us get involved. We really do not know what they are afraid of, they probably think we will scare the tourists away or we will rob. But we honestly do not have bad intentions at all; all we want is to earn an honest living. The tourists might even enjoy our company and our curios too, who knows?  We are very hardworking and innovative. We can make baskets and curious that the tourists may like but the park does not give us that opportunity to mix or just meet the tourists so that we can sell our goods to them.  The park is good to us. Our children have the opportunity to know all kinds of animals.  When CAMPFIRE started, we used to benefit a lot in form of cash, ward offices, schools and many other things, but now we are not getting anything, the council is the only one benefiting. Actually, getting money from CAMPFIRE has become a thing of the past. We are in the second year now without getting a single cent but the hunters are still coming as before  The park staff are slow to respond to complaints especially those that require urgent action like presence of wild animals in our fields. We end up killing the animals because we will be worried about our safety and belongings.  The park staff are very good at ignoring people. Many cattle have to die first before they can respond to our calls. It’s good that they communicate through our leaders because they can’t possibly talk with everyone, but their communication is rather slow.  If you lose anything for example crops, livestock or even property, the park does not compensate you at all.  It is good to conserve wild animals for tourism which brings foreign currency  **FGD 3**  We are not happy here, and our relationship with the park is not even good at all. We always hoped we would benefit through employment in the park but now we see that it is never going to happen, they only employ very few of our people just so that we don’t make noise. But we can see clearly now.  We are happy with the communication though. Although the park communicates with leaders only, our leaders tell us all that need to be done so eventually we get the message. It’s all good. But that alone cannot be enough; the park is reluctant to teach us about animals and tourism. But we know the reason though, they don’t want us to be successful, they think they wouldn’t be able to do what they want with us if we know better.  The park staff treat us like some dump people. We know very well that we are not supposed to kill animals or cut trees illegally because they will get finished, but if we do it, it’s because the park is driving us to, if only they can do their part, then we will do ours. Instead of them harassing our husbands and children, they should try to go to the root of the problem; they should give us our share of the money from tourism now so we are also able to survive.  We want to participate and benefit from tourism, but we don’t know how to. The park is not helping us much.  The park does not help us in any way. CAMPFIRE used to help us a lot but not anymore. We are all alone now, with no one to help us.  Parks staff do not allow us to access resources found in abundance in their park, this is not fair considering the fact that we also play a part in conservation through providing them with information related to poaching incidents in their area, for no return. For example as women, we need to feed our children every day, so during summer seasons, lots of mushrooms grow in the park, we request them to give us access to harvest them, but they do not allow us with the justification that its food for their baboons. This shows that they do not have heart towards humans, what surprises us is that you find them harvesting for their own consumption.  The rangers are cruel, whenever they suspect our husbands or children of poaching from their areas, they come to our homesteads and assault them in front of us and our kids, a situation which is very unfair to us, at times they even beat us, in a bid to force us to provide them with information on the whereabouts of our husbands or children when they do not find them home. For example one of the women at one time attempted to kill herself after being assaulted by these cruel rangers. When the park officials arrived at her homesteaded looking for her husband who was suspected to have poached an elephant in the park, they only found a woman alone, and they asked the whereabouts of her husband, the woman simply told them that she was not aware of his whereabouts, then they started to accuse her of hiding the information and started to beat her up. Feeling humiliated and embarrassed the woman just got in the house and drank poison. Fortunately we quickly saved her life by giving her traditional medicine.  The park is good to us local youths and women, they allow us to enter their park and fish for free three times a week. This helps us to feed ourselves and our families.  The animals are good for us and our country. If only we could share the proceeds equally, everyone would play their part in conserving the animals. The problem now is that only one part benefits. If we ask workers from the park they always say money from tourism is Government money. We then wonder who this Government is for, they use money that comes from here for something else and yet we are the ones whose crops and livestock are destroyed by animals.  Wild animals are a great problem to us. They destroy our crops, property, livestock and even kill people at time, and the park does not even care, they do not give us anything even if you lose everything. At least they should respond quickly and help us when we have problems with animals, not what they do now, coming after several hours and they expect to still find the animals there, really?  The park does not teach us about tourists and what we can do to benefit from tourism.  **FGD 4**  The relationship between the community and the park is too bad. Parks staff can also be mean at times as they always accuse us the youths of poaching their animals. As such they are not willing to engage us as contract labourers, and instead they hire their own relatives from rural homes leaving us. This is not a welcome development to us youth. As youth from the producer community, we feel that we should benefit whenever opportunities like that arise.  Moreover, the park staff do not listen to our concerns in most cases, they just do not care, for example last time a woman was trampled by an elephant and left half dead. We approached the park officials and asked them to come and kill the animal, since it was dangerous to the community but they never come.  Although their communication is often fair, the parks officials are sometimes also full of lies, I remember one other time we (as the community) requested to have a meeting with them, in a bid to air our grievances in relation to problem animals, and promised to come, but they never showed up. These animals are a serious threat to us, we even fear for our lives, let alone our crops and livestock. We have lost a lot, and we have received nothing as compensation. The park officials are really cruel.  The parks officials are sometimes generous to us youths, whenever they do staff recruitment, it seems they have a quota for us, local youths because some of our guys around here, were recently employed. We were very happy with that, it showed that the park has got the youths in their minds. We will keep helping them to protect our resources in the hope that they will also do more for us as many of us are still not employed.  We understand that it may be impossible for the park to absorb all of us who are unemployed, that is why are bitter that the park is letting us down. Besides getting employed in the park, we can do something productive you know, especially when we are given the opportunity to participate in tourism, which the park is adamant to give. You know CAMPFIRE is good but is rather kind of rigid. It does not give us that opportunity to explore new avenues and benefit individually, that is why CAMPFIRE should be left for our old folks while we are actively engaged in other forms of tourism. |
| **PA 4**  **FGD 1**  Our relationship with park is not good at all. At times we do functions like fundraising for the school, Heroes Day or Independence Day celebrations and we ask for meat from the safari, but they refuse. One other time they gave us 10kgs of meat, we really felt insulted. How can 10kgs of meat be expected to feed hundreds of people?  Because of hunger people end up killing animals in the safari, it’s not their problem, what can they do? But the way rangers treat the people is not fair at all, do they have to that militaristic? Are there really no other ways of solving problems without violence?  Aah-aah, it is the problem of us parents, our children bring meat at home and we thank them then go ahead to cook the meat, other children sell game meat and we buy, but we know very well that these animals would have been poached. How on earth can we develop as a community?  We get portions of thatching grass from the safari but we are not happy about it because it is not enough and we are not happy with the process. When you are granted the opportunity you cut three bundles of thatching grass, one is yours and the other two you give to the safari. We are really being oppressed, it’s just that we are poor and we have no option.  I don’t think we are ever going to relate well with the safari. For starters, the owner of this farm says it’s his but we say it is our land, our country. So you see we are never going to agree on anything.  The hunters come and they pay a lot of money to hunt the animals, they take what they want and leave the meat behind, but the safari does not even give us this meat that has already been paid for, in actual fact, they sell us the meat at US$2-00 per kg. Who does that?  Probably selling the meat at a subsidised price is a good strategy, imagine the confusion that would be caused if they were to give the meat for free, how would they share it, it obviously would not be enough for all of us.  We get a lot of benefits from the safari. If the borehole is out of order, we get water for our cattle from the safari. We even get skilled men to fix the borehole for us if it is out of order, they get casual labour from our children here, and they help school children with books and pencils in exchange for minor jobs. However, we are not getting real help with our schools for example building teachers’ houses.  They help us with a vehicle when we have important journeys, e.g., during illnesses or funerals and they also help us with a tractor for ploughing our fields.  In as much as we get help from the ranch, we also suffer terrible losses from animal depredation of our crops especially warthogs. The ranch will not hear anything to do with compensation.  Communication is one of the things that are good between the ranch and us. Although sometimes they ignore our concerns, but at least they give us the opportunity to talk with them. Actually, the ranch holds a meeting with community leaders every month where we are free to discuss all our concerns, some which are listened to and some which are not.  Our people have an issue or concern that we feel has been ignored unjustly. We want to be involved in tourism, to meet the tourists and be able to interact with them, that way we can find ways of boosting our selves economically. The managers of the ranch will not hear of it. They say these are not tourists but hunters, they only come during particular seasons and they are very few, they don’t come in groups like normal tourists. But still, we feel they should just not dismiss us like that, these hunters, as they call them, might even like our staff too. Every tourist takes back some form of souvenirs back home, don’t they?  **FGD 2**  We are not happy about this safari. They sell us meat that has already been bought for US$2-00 per kg. In other words they are selling the same animal twice, these people are really greedy.  What we need in this area is to be educated about our rights with regards to this safari. As far as we are concerned, these people are oppressing us.  Neighbours are supposed to help each other in times of need right. We really wonder what type of a neighbour we have here. We are dying of hunger here and they are not doing anything to help us. So what kind of a stupid relationship is this? This is no relationship at all.  Workers from the safari harass us unjustly without enough evidence. We do not know what kind of law is it that they are using, but all we know is that it is not fair at all.  The tourists come with their cars which we always see going in and out, but we do not know anything about these tourists, the safari does not give us an opportunity to mix with them and sell our products to them, so how can we ever develop?  The safari is helpful at times. They often help with a car or a tractor when we need them; all we have to do is buy the diesel which is a fair deal.  If we are faced with illnesses or death, we also get transport from the safari to take the ill to the hospital and back or carry our dead.  Many of our kids are not getting employment from the safari, and this is something we are not happy about is.  A few years ago a number of our children used to get employed with the safari. However they bring a challenge to the safari because they are friends, neighbours or family to the whole community. If they are going to follow up on poachers or even arrest them they wouldn’t be able to that to their family, but their employer will be waiting for results. Then so they just decided to get many of their employees from other areas.  The animals destroy or crops. This time of the year we do not even sleep in our houses but we guard our crops against animals at night. This is hard but it often helps considering that even if we lose everything we will never get a penny as compensation.  Our communication with the safari is the one with a problem. We are represented by our leaders like village heads, unfortunately some of these leaders are uneducated, they can’t negotiate and are easy to manipulate and oppress.  What we want is to be able to communicate with the safari freely and openly so that we can air our views and discuss with them.  **FGD 3**  We have a bad relationship with the safari but that does not mean they don’t do some nice things for us. The safari does not teach us anything especially to do with tourism, and besides we do not even trust them anymore. Maybe there is need for someone who is neutral to come and educate us on conservation and tourism. That way we will make informed decisions in everything we do. You know as women we need to be seen doing something for our families.  As local women, we feel safari people never consider us as human beings, immediately after rain seasons, we approach their offices seeking permission to harvest thatch grass in the ranch, since it is in abundance, sometimes they do not allow us that access or they give us turns where our harvesting will be strictly controlled and monitored. More often the grass that we get under those circumstances is not enough for our needs since we have to give some bindles to the ranch in exchange for that opportunity. Should we happen to enter the safari without their authority to get more grass to finish thatching our houses and they find us harvesting that thatch grass they assault us greatly without considering our role in the community as women.  We are often forced to leave our houses and husbands and guard our farms against animals at night. You surely understand what that means; we run the risk of losing our husbands to other women. What married man can be patient enough to sleep alone for the whole season? The ranch is not helping us at all; it’s more like we are being forced to choose between our crops and our husbands, what a complication it is. To make matters worse, the ranch won’t compensate even a little bit for the losses we make.  The rangers are very rough especially with our husbands and sons. We know sometimes they can be wrong because poaching can never be right no matter what the circumstances, but they don’t deserve to be treated that way. You know, they are treated far worse than criminals or murderers, they are beaten up, harassed, tortured and even given the worst form of embarrassment ever in front of us their wives or mothers and children. These rangers should have some compassion please.  We appreciate that the ranch managers communicate with our leaders, but we feel they also need to take time to come to the people often, because somehow we feel that these leaders fail to represent our interests but theirs.  **FGD 4**  Our relationship with the park is bad. This is because very few people from our community are employed in the ranch; they prefer people from far away. At the end of the day, one has to do what one has to do to survive. Those animals are our only means of survival.  Our community benefit a lot from the ranch though, things like transport, limited thatching grass and tractors for ploughing your fields if you are lucky or clever enough to befriend the owners of the ranch, but all we are saying is that as youths, we need some form of empowerment, something that can help us make our own money and not to wait for handouts year in and year out.  Since the safari is somehow involved in tourism, although of cause it is mainly hunting, but all the same, it should give the chance as local youth to market and selling our products to those international tourists, when they come to their ranch. We are carving different curios, but we do not have anywhere to sell them. We thought the safari was going to present to us the opportunity to sell these products but the case is not like that.  According to our understanding wild animals belong to the safari, what surprises us is that when their animals raid our fields, we ask them to compensate us, unfortunately they are not willing to do so, with the justification that, when wild animals leave the safari they no longer belong to them, and they cannot be held accountable for compensation. If we opt to kill such animals in our communities they arrest us or beat us to death in the name of wildlife protection. Who can be crueler than that?  The ranch’s participation in community development is just next to zero, whenever they is a function within the community, they do not contribute anything at all, if we ask them to contribute they just give us heap of excuses which only shows how mean they can be at times.  We appreciate that the ranch owners/ managers often engage our leadership to get wind of things, but as the youths we need our own audience. The ranch owners need to hear us out so that we can reason together. |
