# Supplementary material for: Protected area staff and local community viewpoints: A qualitative assessment of conservation relationships in Zimbabwe
Source: PLoS One. 2017 May 19;12(5):e0177153. doi: 10.1371/journal.pone.0177153 (PMC5438145; doi:10.1371/journal.pone.0177153)
Supplement: S2 Table — (DOCX) [file pone.0177153.s002.docx]

**S2 Table: Interview Data Four Protected Areas (PAs) Transcribed Verbatim**

| **PA 1**  **Interviewee 1**  Our relationship is not yet good but I can say we have reached a professional level of acceptance, we have had a situation where the community needed to know the programme we are doing, and we have done fairly well. We are starting to get involved into lots of public orientation though I still think a lot can be done to better the relationship.  I think our main problem is that the communities are expecting everything from me, money, tractors to help them grow their crops, borehole drilling and dam construction, I have a monthly allocation of 50 litres of diesel for using on community requests, which obviously cannot be enough for all of them.  The community is also expecting benefits from us in the form of money, however corporate responsibility happens where there is profit.  What I do know is that the resources in the park is for the benefit of the community , but the resources should self-sufficient first before its distributed to the community, currently we are running at a deficit, so once the program starts to be in full force the community will benefit. At the moment we do not have money to give to the community.  There are developments that are taking place that are directly benefiting communities, we are fixing roads every time that are within the local area. Local schools are visiting the park at a low cost, these are fringe benefits.  There is influx of clients here so the community should basically benefit by selling their products to these clients. I know (musika) marketplace often visited by clients where they buy curios. In my view benefits should be given to the communities living adjacent to the park, for example borehole drilling is a good thing for people to see benefits, because water is important to every household, one borehole can serve 10-20 households. As such we are proposing to drill about three boreholes per year.  The main thing is, the communities should understand what real the project, I am proposing to drill three boreholes for the communities. We as management do not have clear direction as to how the communities are going to benefit.  But at least the communities are benefiting something because all casual labourers are local even, some permanent employees are locals. But compared to the total number of employable local people, very few people benefit from employment in this park. Whilst we are trying our best, most the people do not seem to be satisfied. However, this is understandable; everyone wants a piece of the cake which can never be enough for everyone. But what can we do?  Communities should learn to be resourceful now. They should put lodge at the boundary, so that they benefit, it’s simple enough to achieve, I can go and tell them to do so but they first ask me if I am going to give them money to do so.  Communities complain a lot about almost everything. But everything starts with good communication right? Traditionally, there have been awareness campaigns exercises organised from the parks headquarters in Harare, where they could come here and talk to the local people about the importance of wildlife.  Now we also have a programme together with police where we are going to see the chiefs, District Administrators and everybody in local communities, in order to start streaming from top to down, so that all individuals within the community are much informed about the Parks operations and project.  We are doing awareness campaigns where are giving fliers. In the first two years we gave 2000 fliers to the local people, we even hire people from local people to go and speak with local people.  Poaching is a problem here. People either poach for subsistence or for sale. The problem is that there is limited capacity within the communities in terms of farming inputs which restrict them from realising better socio-economic benefits from crop production. Cash crop farming alone is thus not a very viable livelihood option for the communities hence the need for heavy reliance on wildlife resources.  Gold panning from local people and boundary disputes are also other problems that we face. We have got situations where cattle have been grazing in the park for years, now that we have put fence to stop cattle entering, we are now facing resistance from the local people.  From our park we have cheetahs going to the communities killing goats and elephants raiding crops.  Local people have got no understanding on the value of wildlife, they kill a kudu and sell its meat at $100, my understanding is that, a sable is worthy about 15 cattle. If the community gets the real value of wildlife they will stop cattle farming and start buffalo farming tomorrow. A newly born buffalo is worth $3000, whereas fully grown cow is worthy $500. A fully grown buffalo is worthy $12000, and if local people lose a little or everything from crop raiding by buffalo or elephants, they would still get benefits if were in to wildlife rearing. One buffalo can compensate the whole community. However this information needs to be imparted to the community to understand the value and know the benefits of wildlife conservation.  This joint venture project is benefiting parks, so Bindura council should approach government and say parks should pay back to the producer community a certain percentage.  We have a situation when elephants and cheetahs that were brought here by the project go and raid community crops, in such situations we compensate the affected local people. I am moving cheetahs from the project area to avoid problems. The benefit of having the cheetah here against its damage to the communities is not viable.  I know the community wants to be involved in everything we do, but I assure you we often involve them, especially when it concerns them. For example, when we did environmental impact assessment, we consulted the local communities in order to have their input on the project.  **Interviewee 2**  At the moment the relationship is unpredictable because there are some conflicts, especially the issue of boundary sharing, some villagers say they have rights to enter the park. We ended up consulting the rural district to intervene on the issue, and the matter is yet to be resolved.  First of all we have to deal with political issues, because all our issues end up being political.  There is need for resolving the issue of boundaries between us and the communities; it’s an issue causing development in some situations.  Local people settled along the buffer zones, and left a thin line between the park and the community area. Because they removed the buffer zones it is now difficult to manage the fires, from the local communities.  Livestock encroachment is no longer an issue because we erected a fence, and we try to keep our buffalos as far as possible from their livestock because we know that they might transmit diseases to livestock, a situation that might result in fierce battle with the community members.  On another note, our relationship is not that bad. The communities are very supportive sometimes. In as far as our operations are concerned and security, we get information on poaching activities from local community.  Moreover, sometimes we ask the input of the local people on the community development project, at one time we went and consult them about the project we wanted to do for them. At least we are trying, this counts for something right.  But I do recognise however that traditional knowledge has not been well included in wildlife management system. It has been ignored at the expense of scientific knowledge.    One of the main reasons for an uncertain relationship between the communities and the park is the issue of benefits. Communities are not benefiting much from park; they are yet to know what is happening, so there is need for public awareness campaign to educate them.  Right now the major benefit for the locals is employment, especially contract workers are employed from the locals. Basically from this side we employ from Madziwa.  However, the communities expect much. First, they expect us to build a school, to provide water boreholes and road maintenance. Some local leaders approached us requesting such services but unfortunately we do not have funds for those developments until such a time when the project becomes self-sufficient.  But all the same, there is need to allow local people to access the resources in the park at a regulated level, because if we allow them to access without control we end up in deep trouble with future generations.  The Parks and Wildlife Management Authority does not have a compensation scheme for losses from wildlife depredation. As a park, we do however, sometimes compensate in monetary form, if the wildlife destroys livestock or raid crops, depending on the availability of funds.  At the moment the compensation the villagers are getting from us is fairly good because we agree at a certain figure of compensation.  There has been some disturbances largely due to several factors, one being mistrust, when wild animals raid their crops, they think that we are not concerned despite us making efforts to protect those animals. Our response as far as I am concerned has been quick. Right now we don’t have liaison officer; senior wildlife officers and senior rangers do the awareness campaigns.  We should carry out more meetings with local people, and educate them other issue of joint venture and its benefits to them.  Another problem we face is that some local community members want to access park through undesignated points.  But the major problem is between what the community values and what we value, it’s difficult to local people to understand if their chicken is eaten by a cheetah, they place the value on the chicken not the cheetah.  We trust the locals, but there are some who would want to jeopardise the project for example, we have locals who do poaching and at the same time we have some people from the local community who give us information on poaching.  One more important thing, illegal gold mining is in Gwetera, Umfurudzi and Mazowe is a problem. Mining is a serious problem here because most of the people around park survive on mining; they spend most of their time doing illegal mining.  I think another source of conflict with the communities is the issue of tourism. There is not much involvement of communities in tourism but in future we will open up, we need locals to bring their artefacts and sell to the tourist who visit this park.  **Interviewee 3**  Our relationship is not so good. We had to stop erecting fence boundary in some sections because of conflicts with other section of the community. They view the project as something new, which is meant to prevent the community from accessing the resources in the park estate.  Besides the community always planning to bring down the fence erected as boundary for the purposes of poaching and as a form of resistance, the community does not benefit the park in any way.  Some local people are changing the park boundary moving it inwards, for their benefit, while some are settling close to the boundary.  We often face resistance from the arrested locals. Plus, we impound the local people’s cattle when they enter into the park and ask them to pay a certain fee. Although this helps us reduce livestock encroachment into the park, it does not go well with the communities.  There has been is an increase in poaching after game introduction by pioneer, the locals think that parks is now being taken over by foreigners, so they need to be educated, though some understand. It’s difficult to end poaching here because local people do not have gardens, so they frequent the park hunting and poaching, they can even go to an extent of breaking the fence.  We are employing local people as contract workers, its good in some instances but bad in some. During the time of their contracts they often scout for hot spots, so it’s a problem because having information about hot spots exposes the animals to poaching. Sometime when their contracts expire, these contract workers may then become poachers.    However, some casuals are great; they provide us with information on the poaching activities in and around the park that we use to apprehend the poachers.  One major problem we are faced with is that at the moment we do not have anything in place for the communities. Actually, from its creation this park has done nothing to the community. This is causing bad relationships since communities see no value for the park. If we had money we could drill boreholes for them, so that they appreciate the importance of wildlife.  Elephants raid crops from the local communities and the cheetah went and ate goats in the communities, and the people killed it in retaliation. The problem is that Parks and Wildlife Act does not have provision for compensation in the event that someone has lost crops to wild animals. However, sometimes compensation is done to the local people when their crops have been raided by introduced animals like elephants and cheetahs.  The other thing is that our communication with the park is a bit on the weak side, we used to have personnel from Parks Headquarters, who would come and carry out awareness campaigns, however these days they are no longer coming. People should be educated about wildlife importance always because some local people lack knowledge in terms of wildlife conservation. As a result, I don’t see anyone from local communities helping in conserving wildlife, we have to do it ourselves.  One other problem is that the locals think they are not being considered as human being because they are not usually consulted in developmental projects by parks, especially those that do not concern them.  Maybe if the communities were to be involved in tourism things would be better. Right now the communities only think of the park in terms of poaching and invading the park.  **Interviewee 4**  Our relationship with the community is bad. This is mainly due to lack of benefits especially denied access from park resources. The community is supposed to benefit from the joint venture through money, when hunts are carried out, and through buying game meat at a very low price, to reduce poaching, that will help them to be understand and protect wildlife.  The thing is, I do not have information on the projects being done now to benefit the local communities but what i know is that we assist them with knowledge on rearing fish. During holidays like Heroes days local leadership approach us requesting for meat for their functions and if the head office approves we provide them with it. The meat. For example at one time we were approached by Madziwa teachers college requesting for the meat for the graduation ceremony and we provided them.  However, local people expect us to maintain roads, dams for them for free, since they are close to the park boundary, which I am sure is very difficult for the park.  Although the community does not always benefit directly, we have an interpretation team from our head office that move around schools teaching school children about the importance of wildlife. Also the school children come for game viewing at a subsidised price and take pictures of the wild animals.  One of the challenges we face is that we do not spend more than three months without receiving a poaching report; some even buy dogs for poaching and the same happens with gold panners. We do not frequently arrest gold panners though; we caution them at times and advise them to desist from activities that destroy the environment.  But communities who value wildlife provide us with information about poaching activities in the park, for example in 2000 some poachers from Chinyengetere killed six sub adult sables, through the information from the community members we managed to arrest the perpetrators. |
| --- |
| **PA 2**  **Interviewee 1**  Our relationship with the surrounding communities is bad because we have different interests. Communities surrounding the park estate used to reside in side park before its creation and during that time they used to rely on the resources from the parks. Therefore creation of the park restricted them from accessing such resources.  I should admit our relationship has also been disturbed by the erection of electric fence. While community access to park resources may have been reduced, human-wildlife conflict was reduced too.  Although we do not have a framework to assist he local people and no project is there now to assist the communities because of lack of funds, communities do enjoy some benefits. The major benefit to the communities is through employing them as causal workers for short periods of six months to eight months. We set aside a certain number of local people whom we engage as contract labourers every year as a motivator to stop poaching.  Moreover, the presence of the park has some opportunities for local people who are innovative in making artefacts; they sell their products to tourists who visit our park. So then socially people can benefit through social interaction with foreign clients.  Some local people value the benefits they receive from us even though they might be not tangible.  One other problem we are noticing is that communication with communities is not all good, even though at a certain level, the community liaison officer communicates with local people through traditional leadership or facilitates meetings between the park and the community. Our relationship with local leadership is great, if local people have problems with us, they send their leadership to come and resolve it. Moreover, our liaison officer carry out training programmes especially to the school kids, so that when they grow up they have sense of conservation in their minds.  Poaching issue is affecting us, we don’t have problem with poachers from far communities but instead locals are a problem; we have been arresting these local people frequently and asking them to pay fines or taking them to police for prosecution. We use parks tools to arrest the poachers, if they go against parks laws such as Parks and wildlife Act. Although they benefit through CAMPFIRE, local people are not happy with problem animals in their area.  We carry out several awareness campaigns to educate local people about the importance of wildlife.  The communities might also not be happy because we just run our show; we don’t include local people in decision making.  **Interviewee 2**  Our relationship with local people is good and even now after the land reform we are still good because even though during the land reform there were some conflicts, those who wanted what they want we gave them since its government property.  Although they lost grazing pastures, I would say that the local community were not affected when they were moved out of park estate because the soil in the park is not very good for farming, compared to the areas they were settled when they were moved out of the park. The communities only need to understand this.  The community enjoys some benefits from the park. Using some control mechanisms, we allow them to harvest natural resources, like thatch grass, we also assist them with fuel, vehicle, game guides to go and harvest the thatch grass.  Moreover, we give them the chance to enter into park and honour their ancestors every year.  We expect the local communities to improve their understanding of wildlife, since they are benefiting from money and other non-monetary things through CAMPFIRE and meat from animals hunted in their areas.  We expect the locals to reduce veldfires, even though veld fires were reduced by erection of fences.  We expect communities to assist us in wildlife conservation through work with us  We have a problem with human-wildlife conflicts though. We cannot quantify the problems of human wildlife, but all I know is that they are many because elephants destroy crops regularly during summer season. However the local people should be are happy when the elephants enter their area because of CAMPFIRE; they know that they will harvest that elephants for huge benefits.  If there is problem elephant the local community engage local professional hunters to shoot the elephant for them, in return for that the communities benefit meat and money. We used to have problems with communities over the erection of fence on the boundaries of the park, but however the fence was erected with CAMPFIRE in mind, areas with low wire height are meant to allow animals to cross into the community for the purpose of CAMPFIRE.  One challenge with human-wildlife conflicts is that we have a zero compensation scheme to those whose crops have been raided by the elephants. The argument is that if an animal leaves the park estate it no longer belongs to the park and vice versa.  Communication between us and communities is transparent and also tiresome. We do workshops, meetings, discussions and if there is problem we try to resolve them amicably. As the community liaison officer, my work involves moving around local communities educating local people about conservation issues and CAMPFIRE.  On a darker side there is friction between the communities and PA staff which is mainly caused by poaching and the consequent arrests that are made thereafter. Poaching for subsistence consumption and siltation is a real problem we face from the locals. Local people’s agricultural practises result in sand siltation in water systems, like in Hippo valley and Triangle; their activities are causing river siltation.  Fertilizers being applied in the fields are affecting aquatic systems and introduction of alien plants, as such there is a programme in Limpopo where people move along the river every year removing invasive plants.  Another problem is livestock encroachment in some areas of the park because there are no grazing pastures in local areas, so the locals herd their animals in the park and when those cattle leave the park estate, they would have contracted wild animals’ diseases like heartwater. My homestead is close to Parks, I lost two of my cattle because of that disease just recently.  We work with Police, EMA, AGRITEX and veterinary services. We come together and disseminate information to the communities about effects of grazing the cattle in the park. As a way to minimise cattle encroachment, we impound the cattle if found in the park and ask the owners to pay punishment fee, the aim is to reduce encroachment not to push them out. But obviously, the community may not understand this.  Deep down I actually think that the communities value wildlife conservation but because of grazing land which they are now restricted from they complain in some situations because to then it the park values wildlife more than people and their livestock.  The community has a lot to be grateful for, we respect them and we value communities input, as a matter of fact, we plan together with local communities, including the chief, Rural District council and conservancies.  We also incorporate community cultural events in our programmes and at our reception here we sell curios for the local communities.  **Interviewee 3**  Our relationship with the community is bad. During land reform there was a problem, where people were resettled in the areas where wildlife was located. Settlement reduced the home ranges of animals and this resulted in animals being pinned in one area. And some of these resettled people took advantage of that and illegally kill the animals around in their locality.  Communities also experience problem from crop raiding from wild animals during harvest seasons. Local people complain if we fail to react in time, some can even use their vehicles to come and pick us to react to those problems. It is difficult to control human wildlife conflict in the resettled area since some of these people occupied areas close to the park boundaries and even inside the park boundary.  Fencing is preventing wild animals to cross into communities. Also fencing is preventing cattle from encroaching into park estate; however at the moment in some areas the animals are still access grazing land in areas where there is no fence.  If there is a report of problem animals we react quickly, we can assess the damage but we are not able to compensate them because of cash shortage. Also there is no law to enforce compensation. The communities are aware that Parks is not liable to compensate them in the event of crop raiding from wild animals. Some understand and accept the situation but some do not.  Besides these problems, we are employing community people in thatch grass harvesting, road maintenance and fencing. Mostly we employ women for thatch grass, and this is done in relation to the area where the project of thatching in the park is done. Also some of the permanent employees are from the local communities. Most of them are not permanently employed because of poor educational qualifications.  Parks do not have a market place where communities could come and sell their products instead the communities should locate a place outside park estate where they can sell their products to tourist on the way out of the Parks.  Parks is afraid to open a place within the Parks where locals could come and sell their products because of security reasons which includes poaching if locals are to be given free access into the park. Also the locals will be exposed to the dangerous animals when they come into the park.  However, local operators in tourism sector employ local people for example Bush camp safaris, employed local people.  Some Communities sell baskets to operators and clients who come and visit the park. Unfortunately we do not have provision to market local people products.  Due to security in terms of poaching and theft reasons, it’s difficult to encourage local communities to come and sell their products to the tourist within the park estate, also I have never seen anyone from the local communities approaching parks requesting for such a favour, neither have I seen anyone from Parks advising the local people to come and sell their products here to the tourist who visit.  Although our relationship is not all that rosy, as parks we try by every means to increase awareness campaigns and we expect council to increase awareness campaigns also in their areas to the locals and inform them that some of the properties in the their communities is coming from wildlife harvesting, for example the grinding meal in some of the community were bought out of CAMPFIRE activities. As such communities should be well informed about such developments. In return that will give them sense of ownership to the wildlife that occurs in their area.  We also consult locals if they are issues concerning them, which needs to be addressed. For example if the is an increase in animal poaching we approach them and deliberate over the issue, in a bid to find a solution. Whoever if it an issue concerning contravening parks laws, we just effect law as it is.  Above all we value traditional knowledge from the community.  Actually, the main source of our current problems with the park is the fence. Cattle from local communities are encroaching into the park estate for the purpose of pastures, since there is limited grass in the community areas. When those cattle’s encroach they are vulnerable to diseases like anthrax. As a counter measure we erected fence in areas we experience such problems of encroachment.  One of the problems that the communities face that increase their dependency on wildlife resources is that this area experience high temperatures and low rainfall and as such is not very suitable for crop production. This situation is worsened by wild animal destruction of crops as well as lack of financial resources for purchasing agricultural inputs.  Regardless, we try to communicate with the communities although communication is still limited to community leaders. The liaison officer communicates with local community if there is anything to be communicated between the parties. The communities are totally unpredictable you know, one day you think you are together, they are all supportive, the next day they are totally against you, you organise a workshop for them, they don’t come. However, giving them controlled access to some wildlife resources like thatching grass, whenever we can is part of our social responsibility, it does’t matter whether they meet these expectations or not.  At times communities communicate to us using letters or through their chiefs or kraal heads. If there is an increase in poaching from local people we hold meetings with local chiefs edging them to advise other local members to stop illegal hunting.  If the locals request assistance we help them were possible, for example we assist the with transport to carry them within the park to carry out their festival such as ***Sila,*** were they enter the park and harvest fish using trapping nets known as *Duwo.*  Over and above all I think the relationship can be fixed but it will take some hard work. Local people should be educated about utilising their livestock rather than just be boosting of large heads of cattle, without utilising them, instead they opt to enter into park estate and poach wild animals. However it’s difficult to change people’s mind, large heads of cattle is a pride for them, you cannot change them.  If the community knows what is natural resources and why many tourists come from as far as Europe, that will help them reducing poaching and starts to value wildlife.  Some school children from surrounding communities now understand the relevance of wildlife because of educational tours and awareness campaigns from us. |
| **PA 3**  **Interviewee 1**  The relationship is generally good. This is because if there is information about either poaching or any other conservation issues we need from the locals they give us willingly and openly.  As a national park, we contribute greatly towards CAMPFIRE, we share boundary with Nyaminyami CAMPFIRE, as such by virtue of our status as National Park we don’t offer hunting services in the park, but our neighbour, the Nyaminyami, does hunting activities. Therefore Matusadona is working as a breeding ground for CAMPFIRE, all hunting activities being done in Nyaminyami are successful and the trophies from the animals hunted are great.  Our communication with local communities, through their leadership, I can say is generally good, Chief Nebiri at times visits us, a sign of good relationship, last year he requested for an Kudu, for consumption and we managed to hunt one for him and his people. Whenever there is a ward meetings in ward 8, in which we fall under, we are always invited.  Since we are surrounded by communities, during dry season, we experience problems from local women who illegally enter in the park estate to poach some roots called *Manyanya* for consumption. We experience serious dogs and wire snares poaching from local people, most of the elephant poaching is from people from Gokwe, Chitekete. Dealing with such problems always result in some form of conflicts.  We carry out awareness campaigns regularly, and we take advantage of chief’s weekly gathering with his people, if we are free, we go and address people on the importance of wildlife to them and CAMPFIRE programme.  People from Nyaminyami have better understanding of wildlife because of CAMPFIRE programme which has been in their area for years now and the developments it has brought to the communities. As such we don’t face much resistance when we carry out awareness campaigns in the communities.  We consult local communities in issues that affect them, like problem animal control, we seek information from local leaders to have a holistic approach in fighting poaching in and around park estate.  In the event of a problem animal in the communities and we fail to react in time that results in weakening our relationship with the locals.  The locals benefit from employment in the park. Well, I do not have the actual figures of the local employed people at hand, but what I know is that the highest number of the employees is from the surrounding local communities. Casual workers are also employed from the local communities as a motivator and have them appreciate wildlife.  In as much as the communities are benefit, we feel they should also benefit from tourism. However, the park has not done much to facilitate this. Similarly, I have also not seen any local people coming to sell their products to the tourists or coming to request to sell their products to the tourists. I know the communities want this and if this were to materialise, it will be a welcomed initiative. I cannot say there is no opportunity but it’s just that there is lack of knowledge; the communities need to be educated so that they develop a better understanding in terms of wildlife conservation and tourism activities.  **Interviewee 2**  The relationship is generally good, with few conflicts of course. Our relationship with local people is not bad but to those poachers yeah, it’s not good at all. At the moment poaching is not serious, local people harbour and provide information to the foreign poachers, to come and poach their own resources.  The people benefit from CAMPFIRE so it’s all good. CAMPFIRE programme is benefiting a lot, since Matusadona is a breed ground, animals breed and move to the local communities and be hunted for the benefit of the local communities.  To strengthen our relationship as well as for conservation awareness, we carry out workshops to educate people on the importance of wildlife. Those not involved in poaching do understand the situation.  **Interviewee 3**  Our relationship with communities is not all that good. We are experiencing substance poaching from local people, who take advantage of knowing our staff numbers; if they are aware that we are off to shopping they get in the park in numbers and illegally kill small game. Some of the rangers here are from local community so they provide information about the station deployments and this will result in high poaching.  Some of us we are living in fear of some of community members whom we arrest, or whose relatives we arrest. If they have information that you are the one who arrested their relative, they will make sure that they come after you and beat you up thoroughly.  We are experiencing illegal harvesting of trees for fire wood from local people; they are cutting down trees to use for cooking and burning their bricks during moulding.  But the communities can also be good at times. They give us with valuable information on the cases of wildlife poaching. In some situations local people arrest these illegal poachers at one time, they arrest three ivory poachers whom they had found carrying two elephant ivory. They arrested them and bring them our office.  We are also good to the communities. By virtue of us being close to the local community, it means we are able to react quickly to their request such as problem animals; we do not take time before we react to those problems.  The locals also benefit from the park through employment. Mostly contract workers are employed from communities surrounding us. We try to motivate the locals to develop love by engaging them in temporary works. If opportunities arise, they can be employed as permanent employees.  We also help the locals to maintain roads where possible.  We also provide locals with meat whenever we can when they are holding state functions.  We allow them to enter the estate to view animals at a low price.  However, our communication with locals especially us rangers is not that all great, they consider us a stumbling block to them, in their quest to enter and hunt in the park and also they accuse us of beating them up if we find them poaching.  We are always at war with some of these people and they do not want to associate with us in anyway. Even communication with these people can be difficult so mainly communication is limited to the leaders. However, we as the park do not have bad blood against them, and that will not stop us from doing our duties.  For example we carry out awareness campaigns which are usually focused towards the school children, with the intention to impart knowledge to them whilst they are still young, and we believe with that they will be able to utilise the knowledge when they are grown up. |
| **PA 4**  **Interviewee 1**  Our relationship is reciprocal. One time the Rural District tried to evict us at some point from this farm, and they send their guards here and occupy the place, fortunately the community members teamed up and chased the council guards away. I like these people and wish them well, they do not steal from us.  There is one thing that the communities do not seem to understand though. The tourists that come here are mainly hunters who have specific hunting seasons and travel in groups. I would love the communities to benefit from tourism but these are not the kind of tourists they need for that. They won’t help much and so we can’t possibly help the communities there.  The community has got our support, we help them with resources. We use my vehicle to move them from one place to another, ferry sand for them, support football teams, collect their fertilizers and seeds for them, help them with transport to church and political functions. We bring their food relief and employ locals.  We allow them to cut grass for thatching in my farm, but that has to regulated, women who want to cut grass register with me, we carry them to and from cutting grass, after cutting we get one bundle of grass and the other two will be taken by the cutter. We carry the thatch grass not possibly to their homes because we might spoil our tyres, so we get as near as possible.  The locals are really appreciating our support very much; they often send us thank you notes.  Our communication is actually good. Some people call us direct; we always make sure to respond accordingly.  **Interviewee 2**  I think the relationship is pretty good. We have good communication where we have scheduled meeting with the local leaders at least once a year. We try to bring the headmen from the villages surrounding boundaries to our house to have a meeting and discuss problems which they are having and problems we are having, and we work on the solutions together. Everyone’s issues will be tabled before we have general discussions on the environment and weather. This is important to farmers. We also explain the dangers of poaching and what’s going around us.  Lots more interaction with the locals is needed. We (the management) spend lot of time with local leaders and driving them places, touring their crops, we are interested in crops, making comments, looking in their fields, dams giving the people comments. We try to explain in a manner they understand. Good constant communication with headmen is what we need to maintain a positive relationship with the communities.  Some of the locals do understand, especially older generation understand that they do not have to poach, it’s like stealing somebody’s cattle. The younger generation do not seem to understand the real picture.  We used to have little poaching before and there was little snaring. We are however experiencing increased poaching occurrence and tick-borne disease which we never had before from the local people’s cattle.  We extend a lot of benefits to the community, for example, we donate lots of meat to primary and secondary schools, we weld their scotch cuts, battery recharge, bicycles fix, doors fixing and engine repair. If they want to use our tractor, or vehicle we ask them to buy fuel, we believe that they should not get everything for free. Actually we are spending more time fixing their engines than before.  The locals also benefit from employment. Most of the employees are from the local communities.  **Interviewee 3**  The relationship was bad during resettlement time, now the relationship is improving, we are now interacting greatly. We even attend political meetings with them.  Before land reform animals could move freely in and out of the park, these days if they move out people will poach them. We have serious poaching from local people at a rate of 4 to 5 cases per month which are recorded, mostly they kill warthogs. They also kill kudus using wire snares.  Poaching is a problem. I do not think will be able to solve it, because it’s everywhere, it’s not going to end. Elderly people understand the importance of wildlife but young ones need to hunt for the purpose of money.  Very few understand wildlife conservation; they only want to kill the animals for consumption. If poaching increases, we consult the local people, on the likely cause and the solution to the problem.  Regardless of this, we assist the locals with vehicles to carry their deceased for burial; we provide vehicles to carry sand to build their houses.  We employ local people to work for us, unfortunately some are not willing to work in farms instead they want in town.  **Interviewee 4**  Our relationship is not good, especially to us game rangers, whenever I go outside l don’t feel free, and some people shout at us and also do not greet us.  We try at times to improve the relationship, we need to work together with the locals and we want them to stop poaching.  Before resettlement we could record a single poaching incident per month, these days we are now recording those incidents on a weekly basis.  As rangers we are always at war with local people when we go out to beer halls or when we are just going to bus stop, one has to be ready to fight someone, this happens to us rangers only. Local people feel that if we found out that one of the locals has poached, we should not follow them at their houses, we just have to ignore the issue.  However, the communities benefit a lot from the park. At times we try to meet locals to solve the issues of poaching, even try to give them meat for free, as away to move them from poaching. We often give them about 20-30kgs of meat when they are having functions in their area. We offer them transport to areas as far as Nyamandlovu and if they are going in large numbers we give them a big truck. If local people have a broken borehole, we go and help them fixing it for free.  40 % of the locals are happy for what we are doing to them, though some are not.  If there is a problem we can talk to the headmen only, especially those who hate poaching, and they quickly understand us.  Bush pigs are a problem to local people’s fields, but we chase them, we encourage communities to put fire during the night at their fields, if warthogs raid community fields we tell them that they should not kill them, instead they should refer the issue to our offices for action. The only issue which the communities cannot accept is the fact that we cannot compensate them for their losses. But this is not our fault, even Parks and Wildlife Authority does not compensate people for losses due to wildlife depredation. |
